# Microrobotic swarms for selective embolization

**DOI:** 10.1126/sciadv.abm5752

**Published:** 2022-07-20

**Authors:** Junhui Law, Xian Wang, Mengxi Luo, Liming Xin, Xingzhou Du, Wenkun Dou, Tiancong Wang, Guanqiao Shan, Yibin Wang, Peng Song, Xi Huang, Jiangfan Yu, Yu Sun

**Affiliations:** ^1^ Department of Mechanical and Industrial Engineering, University of Toronto, Toronto, Canada.; ^2^ Shenzhen Institute of Artificial Intelligence and Robotics for Society, Shenzhen, China.; ^3^ School of Science and Engineering, The Chinese University of Hong Kong, Shenzhen, China.; ^4^ Program in Developmental and Stem Cell Biology, The Hospital for Sick Children, Toronto, Canada.; ^5^ Arthur and Sonia Labatt Brain Tumour Research Centre, The Hospital for Sick Children, Toronto, Canada.; ^6^ School of Computer Engineering and Science, Shanghai University, Shanghai, China.; ^7^ Department of Interventional Therapy, National Cancer Center/National Clinical Research Center for Cancer/Cancer Hospital and Shenzhen Hospital, Chinese Academy of Medical Sciences and Peking Union Medical College, Shenzhen, China.; ^8^ Department of Molecular Genetics, University of Toronto, Toronto, Canada.; ^9^ Institute of Biomedical Engineering, University of Toronto, Toronto, Canada.; ^10^ Department of Electrical and Computer Engineering, University of Toronto, Toronto, Canada.; ^11^ Department of Computer Science, University of Toronto, Toronto, Canada.; ^12^ Robotics Institute, University of Toronto, Toronto, Canada.

## Abstract

Inspired by the collective intelligence in natural swarms, microrobotic agents have been controlled to form artificial swarms for targeted drug delivery, enhanced imaging, and hyperthermia. Different from these well-investigated tasks, this work aims to develop microrobotic swarms for embolization, which is a clinical technique used to block blood vessels for treating tumors, fistulas, and arteriovenous malformations. Magnetic particle swarms were formed for selective embolization to address the low selectivity of the present embolization technique that is prone to cause complications such as stroke and blindness. We established an analytical model that describes the relationships between fluid viscosity, flow rate, branching angle, magnetic field strength, and swarm integrity, based on which an actuation strategy was developed to maintain the swarm integrity inside a targeted region under fluidic flow conditions. Experiments in microfluidic channels, ex vivo tissues, and in vivo porcine kidneys validated the efficacy of the proposed strategy for selective embolization.

## Teaser

Magnetic microrobots form swarms and selectively block blood vessels for clinical embolization.

## INTRODUCTION

Collective behaviors widely exist in nature. Compared with individual entities, swarms are capable of performing more complex tasks, for instance, fish schools for avoiding predators (*1*) and insect swarms for building nests (*2*). Inspired by the collective intelligence in natural swarms, a variety of robotic swarm systems have been developed, such as Kilobots (*3*), swarm-bots (*4*), and loosely coupled robots (*5*) to perform targeted locomotion (*3*–*5*), obstacle avoidance (*4*, *5*), and object transport (*4*–*6*).

At micro-nanoscales, agents rely on physical and chemical interactions to form microrobotic swarms, via applying external stimuli, including magnetic fields (*7*–*9*), electric fields (*10*–*12*), acoustic fields (*13*–*15*), optical fields (*16*–*18*), and chemical signals (*19*–*21*). Because of their simple morphology and good controllability, magnetic colloids have been used to form swarms, through developing actuation strategies (*22*–*24*), and conduct biomedical tasks, such as targeted drug delivery (*25*–*27*), enhanced imaging (*28*), and hyperthermia (*29*). Magnetic particle swarms driven by rotating magnetic fields and magnetic field gradients have been shown to move in a bovine eye (*25*) and blood vessels (*26*) for targeted drug delivery. Induced by oscillating magnetic fields, heat generated by magnetic swarms was used for hyperthermia in cancer treatment (*29*, *30*). Different from these well-investigated tasks, embolization is a clinical technique used to block blood vessels for the treatment of various diseases, such as tumors, fistulas, and arteriovenous malformations (*31*). In the present clinical practice of embolization, embolic agents are deployed via a catheter. Millimeter-sized platinum or tungsten coils are used to occlude large vessels (*31*, *32*), while agents such as polymeric particles and gelling solution are released upstream to block downstream vessels (*33*). Because of the low selectivity of these passive agents, unintentional blockage of nontargeted blood vessels occurs, leading to severe complications such as stroke and blindness (*31*, *32*, *34*). In comparison, magnetic particles can be promising embolic agents as they are capable of forming swarms on demand via applying magnetic fields. To achieve selective embolization, the swarm integrity inside a targeted region should be maintained while breaking the swarm integrity outside it, which indicates that the magnetic field strength should be maintained sufficiently high inside the targeted region and reduced abruptly outside it. Existing magnetic micromanipulation strategies are not suitable to achieve this goal because they use either uniform magnetic fields that lack selectivity within the workspace (*9*, *22*–*25*) or magnetic field gradients where the field strength monotonically decreases from the external magnetic source to the workspace center (*26*, *27*, *35*). The attainment of the desired spatial distribution of magnetic field strength for selective embolization demands dynamic planning of magnetic field gradient.

Here, we present an actuation strategy for maintaining the integrity of magnetic particle swarms to accurately block the blood flow inside a targeted region for selective embolization. An analytical model describing the relationship between magnetic field strength and swarm integrity was proposed. On the basis of the model, a dynamic magnetic field was designed to form particle swarms and maintain their integrity at targeted junctions of microchannels under fluidic flow conditions. Using thrombin-coated magnetic particles and the proposed strategy, selective embolization in ex vivo porcine omentums and in vivo porcine kidneys was realized. This work deepens the fundamental understanding of microrobotic swarm behaviors under physiological conditions and represents the first proof of concept of selective embolization.

## RESULTS

### Maintenance of swarm integrity in flow

**Fig. 1.**
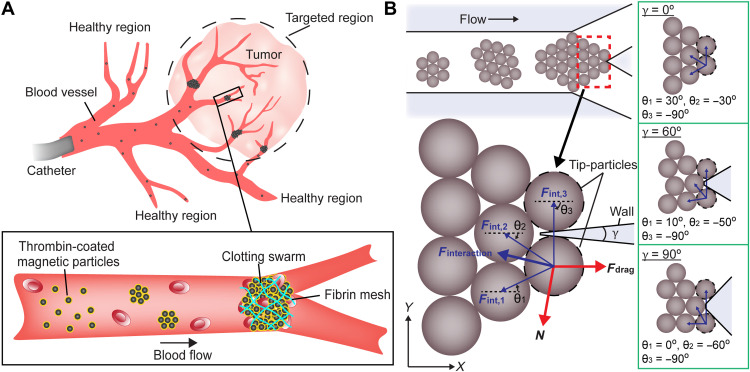
Maintenance of swarm integrity at targeted junctions. (**A**) Schematics illustrating the use of magnetic particle swarms to block the junctions inside a targeted region. (**B**) Schematic analysis of the forces exerted on tip-particles. The brown circles indicate magnetic particles. The black dashed circles denote the tip-particles. The magnetic interaction forces and their resultant interaction force are indicated by thin blue arrows and a thick blue arrow, respectively. The fluidic drag force and the reaction force are indicated by thick red arrows. γ is the branching angle of the junction. θ is the angle between the magnetic interaction force and the *x* axis. The configurations of particles at junctions with different branching angles are demonstrated in the green boxes. Purple regions represent the walls of junctions.

To achieve selective embolization, microrobotic swarms can be generated on demand to block the blood vessels inside a targeted region, as shown in Fig. 1A. Microrobotic agents can be released near a targeted region through a catheter to minimize the required dosage. We used superparamagnetic particles with a diameter of 1 μm, which is smaller than that of red and white blood cells, to ensure that they can be distributed in blood capillaries. The microparticles were coated with thrombin, which converts soluble fibrinogen in the blood into fibrin meshes to trap red blood cells (RBCs) with the particles. The concentration of thrombin on the particles was experimentally tuned (see Materials and Methods), and the particles were actuated to form clotting swarms at the targeted junctions. However, under flow, swarms would be split at the junctions if interactions between particles are weak. Here, the maintenance of swarm integrity in microfluidic channels with physiologically relevant conditions, i.e., branching of blood vessels and blood flow, was investigated. Magnetic particle suspension was injected into a Y-shaped microfluidic channel with 120° branching angles at a flow rate of up to 120 μm/s (see Materials and Methods). A rotating magnetic field with a frequency of 20 Hz was applied to form swarms (fig. S1). When the swarms reached a junction, if the magnetic agent-agent interactions were sufficiently strong, the swarm integrity was maintained. Otherwise, they were split and then moved with the flow. These split swarms were disassembled by flow when they were outside the magnetic field region (fig. S2).

To investigate the relationships between the branching angle, flow rate, magnetic field strength, and swarm integrity, the swarm at a junction is modeled. The particles on the front are taken as representative ones (named tip-particles, as shown in Fig. 1B). Tip-particles are exerted with the strongest constraining magnetic interactions in the swarm. Thus, the maintenance of the tip-particle configuration indicates that the swarm integrity can also be achieved. In contrast, if the tip-particles are disassembled by the flow, some of the remaining particles of the swarm would serve as the new tip-particles with weaker constraining magnetic interactions, which would still be disassembled until the entire swarm is split. In our analysis, the coordinate system is defined as shown in Fig. 1B. In a low–Reynolds number fluid, the inertia of particles is negligible. The forces exerted on a tip-particle in the *x* axis are

$$F_{\text{drag}}+F_{\text{interaction},x}+F_{r}\text{sin}\frac{\gamma }{2}=0$$ (1)

where **F**_**drag**_ is the fluidic drag force, **F**_**interaction,x**_ is the *x*-axial component of the resultant magnetic dipole-dipole interaction force, **F**_**r**_ is the reaction force exerted by the wall, and γ is the branching angle of the junction. The fluidic drag force exerted on a spherical particle is

$$F_{\text{drag}}=-C_{d}(v_{p}-v_{f})$$ (2)

where *C*_d_ is the drag force coefficient, **v**_**f**_ is the flow rate, and **v**_**p**_ is the velocity of the tip-particle. For compacted inner particles within a swarm, their average drag coefficient is less than that of uniformly distributed particles (*36*) and can be expressed as *C*_d_ = 3πη*dk*, where η is the dynamic viscosity of the fluid, *d* is the diameter of the particle, and *k* is a drag coefficient correction factor. The magnetic interaction force exerted between particles can be expressed as (*37*)

$$F_{\text{int,ij}}=\frac{3V^{2}{\chi_{p}}^{2}}{4{\pi \mu }_{0}{r_{\text{ij}}}^{4}}[(1-5{\left(\hat{B}\cdot {\hat{r}}_{\text{ij}}\right)}^{2}){\hat{r}}_{\text{ij}}+2(\hat{B}\cdot {\hat{r}}_{\text{ij}})\hat{B}]B^{2}=c_{\text{int,ij}}B^{2}$$ (3)

where **F**_**int,ij**_ is the interaction force exerted on tip-particle *j* by particle *i*, *V* is the volume of the particle, *r_ij_* and ${\hat{r}}_{\text{ij}}$ are the distance and unit vector between the center of particle *i* and that of tip-particle *j*, respectively, χ_p_ is the effective magnetic susceptibility of the particle, μ_0_ is the permeability of free space, *B* is the magnetic field strength, $\hat{B}$ is the unit vector of the magnetic field, and the coefficient can be expressed as $c_{\text{int,ij}}=\frac{3V^{2}{\chi_{p}}^{2}}{4{\pi \mu }_{0}{r_{\text{ij}}}^{4}}[(1-5{\left(\hat{B}\cdot {\hat{r}}_{\text{ij}}\right)}^{2}){\hat{r}}_{\text{ij}}+2(\hat{B}\cdot {\hat{r}}_{\text{ij}})\hat{B}]$.

With Eq. 3, the *x*-axial component of the resultant magnetic interaction force exerted on a tip-particle can be calculated as

$$\begin{array}{cc} F_{\text{interaction},x} & =[\begin{array}{ccc} F_{\text{int},1} & F_{\text{int},2} & F_{\text{int},3} \end{array}][\begin{array}{c} \text{cos}\;\theta_{1}\\ \text{cos}\;\theta_{2}\\ \text{cos}\;\theta_{3} \end{array}]\\ & =B^{2}[\begin{array}{ccc} c_{\text{int},1} & c_{\text{int},2} & c_{\text{int},3} \end{array}][\begin{array}{c} \text{cos}\;\theta_{1}\\ \text{cos}\;\theta_{2}\\ \text{cos}\;\theta_{3} \end{array}] \end{array}$$ (4)

where indexes 1, 2, and 3 indicate the neighboring particles that are exerting on the tip-particle, and θ is the angle between **F**_**int,ij**_ and the *x* axis. By analyzing the configurations of particles at junctions with different branching angles, θ_1_, θ_2_, and θ_3_ are estimated, as shown in Fig. 1B. Balancing the forces in the *y* axis, the reaction force is

$$\begin{array}{c} F_{r}=-\frac{1}{\text{cos}\frac{\gamma }{2}}[\begin{array}{ccc} F_{\text{int},1} & F_{\text{int},2} & F_{\text{int},3} \end{array}][\begin{array}{c} \text{sin}\;\theta_{1}\\ \text{sin}\;\theta_{2}\\ \text{sin}\;\theta_{3} \end{array}]\\ =-\frac{B^{2}}{\text{cos}\frac{\gamma }{2}}[\begin{array}{ccc} c_{\text{int},1} & c_{\text{int},2} & c_{\text{int},3} \end{array}][\begin{array}{c} \text{sin}\;\theta_{1}\\ \text{sin}\;\theta_{2}\\ \text{sin}\;\theta_{3} \end{array}] \end{array}$$ (5)

where 0 ≤ γ < π. By substituting Eqs. 2, 4, and 5 into Eq. 1, the critical magnetic field strength *B*_critical_ maintaining the configuration of tip-particles in the swarm can be derived as

$$B_{\text{critical}}={\left(3\pi \eta \text{dk}(v_{p}-v_{f}){\left([\begin{array}{ccc} c_{\text{int},1} & c_{\text{int},2} & c_{\text{int},3} \end{array}][\begin{array}{c} \text{cos}\;\theta_{1}\\ \text{cos}\;\theta_{2}\\ \text{cos}\;\theta_{3} \end{array}]-\text{tan}\frac{\gamma }{2}[\begin{array}{ccc} c_{\text{int},1} & c_{\text{int},2} & c_{\text{int},3} \end{array}][\begin{array}{c} \text{sin}\;\theta_{1}\\ \text{sin}\;\theta_{2}\\ \text{sin}\;\theta_{3} \end{array}]\right)}^{-1}\right)}^{\frac{1}{2}}$$ (6)

From Eq. 6, it can be predicted that *B*_critical_ becomes higher when the viscosity of fluid η and flow rate **v**_**f**_ are higher, and *B*_critical_ has a negative relationship with the branching angle γ. When a dynamic magnetic field is applied, the component $\hat{B}$ in the coefficient **c**_**int,ij**_ varies with time, resulting in different *B*_critical_ over time. Therefore, an average value of *B*_critical_ is calculated via numerical simulation.

**Fig. 2.**
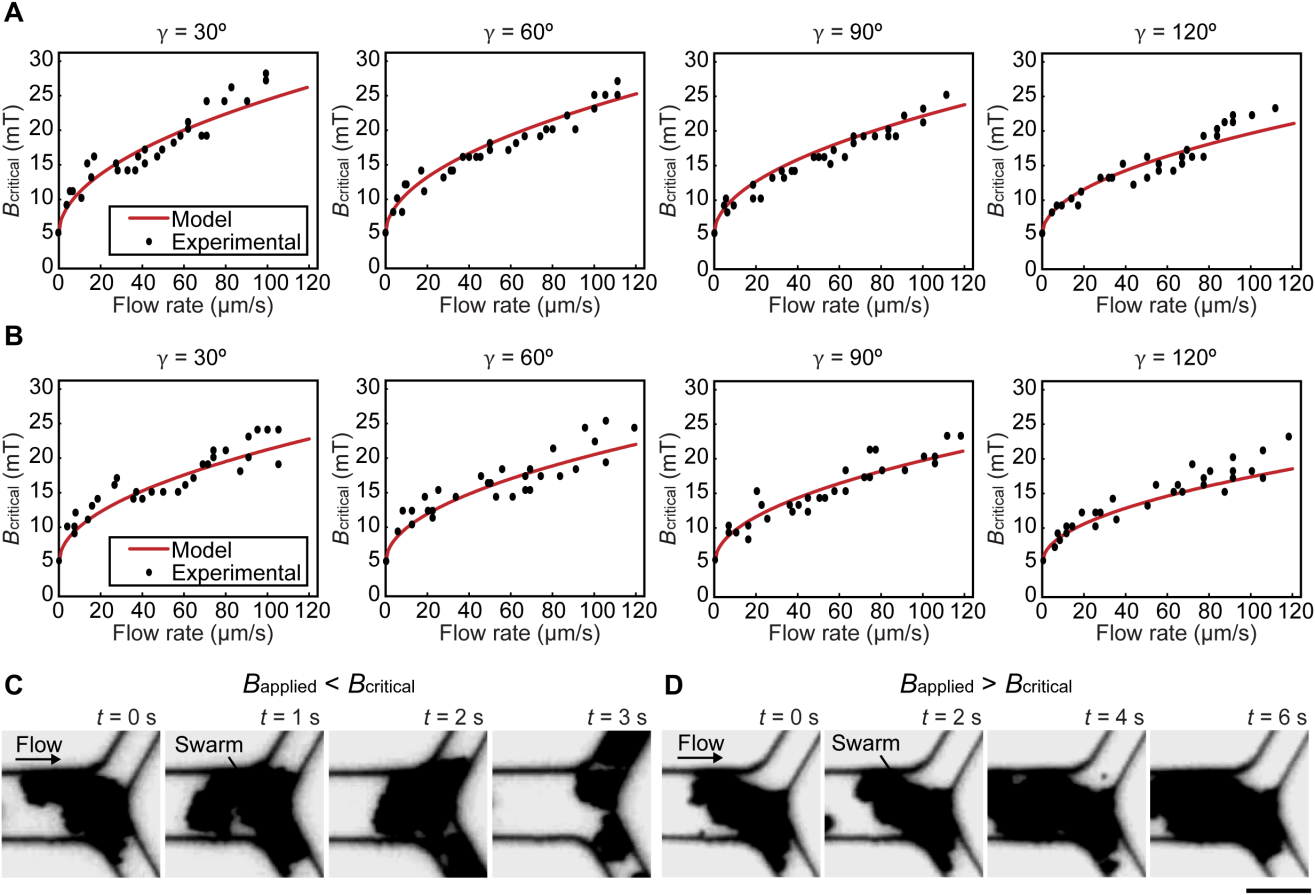
Experimental validations for the model. (**A** and **B**) The relationship between critical magnetic field strength *B*_critical_ and flow rate at junctions with different branching angles γ in porcine whole blood and PBS, respectively. (**C** and **D**) The integrity of swarms when the magnetic field strength applied was lower and higher than *B*_critical_, respectively. Scale bar, 20 μm.

We then experimentally investigated the influence of flow rate, branching angle, and fluid viscosity on *B*_critical_. In experiments, Y-shaped microfluidic channels with branching angles of 30°, 60°, 90°, and 120° were used, which cover the range of physiological branching angles of vascular networks (*38*). Magnetic particles suspended in porcine whole blood were injected into the channels at flow rates of up to 120 μm/s. The flow rates were quantified by measuring the speed of tracing particles in the fluid. As discussed above, *B*_critical_ is the minimum field strength that can maintain swarm integrity at a junction. Figure 2A shows the experimental results (black dots), from which one can see that a higher *B*_critical_ was required when the flow rate was higher, and with the same flow rate, *B*_critical_ was lower when the branching angle was larger. To quantify the influence of fluid viscosity on *B*_critical_, magnetic particles suspended in phosphate-buffered saline (PBS) solution were used [PBS’s viscosity: ≈1 cP versus porcine blood plasma’s viscosity: 1.4 cP (*39*)], and the results are shown in Fig. 2B. Compared to the data in Fig. 2A, with the same flow rate and branching angle, *B*_critical_ was lower when the viscosity of the fluid was lower. The calculated *B*_critical_ values in porcine blood plasma and PBS solution were plotted as the red lines in Fig. 2 (A and B), respectively (see Materials and Methods). Relative errors were calculated by dividing the result differences between the model and experiments by the model. In porcine whole blood and PBS solution, the average relative errors were 8.0 and 9.1%, respectively. These errors can be attributed to the proposed model that was built based on the two-dimensional (2D) model of swarms; however, swarms have multiple layers of particles along the *z* axis, resulting in the change of magnetic interaction forces and thus causing the approximately 9% errors. To verify the generality of this 2D swarm integrity model, we compared its predictions to those of 3D swarm integrity models (see Materials and Methods, fig. S3, and table S2). Statistical tests showed no significant differences among the variances of the models, indicating that the 2D model sufficiently represents swarm integrity. The variation in the predictions of the 3D models also explained for the variation in the experimental results (fig. S3). Figure 2C shows that swarms were split when the magnetic field strength applied was lower than the calculated *B*_critical_ (movie S1), while swarms maintained their integrity at a junction when the magnetic field strength applied was higher than the calculated *B*_critical_ (Fig. 2D and movie S2).

### Actuation strategy for selective maintenance of swarm integrity

**Fig. 3.**
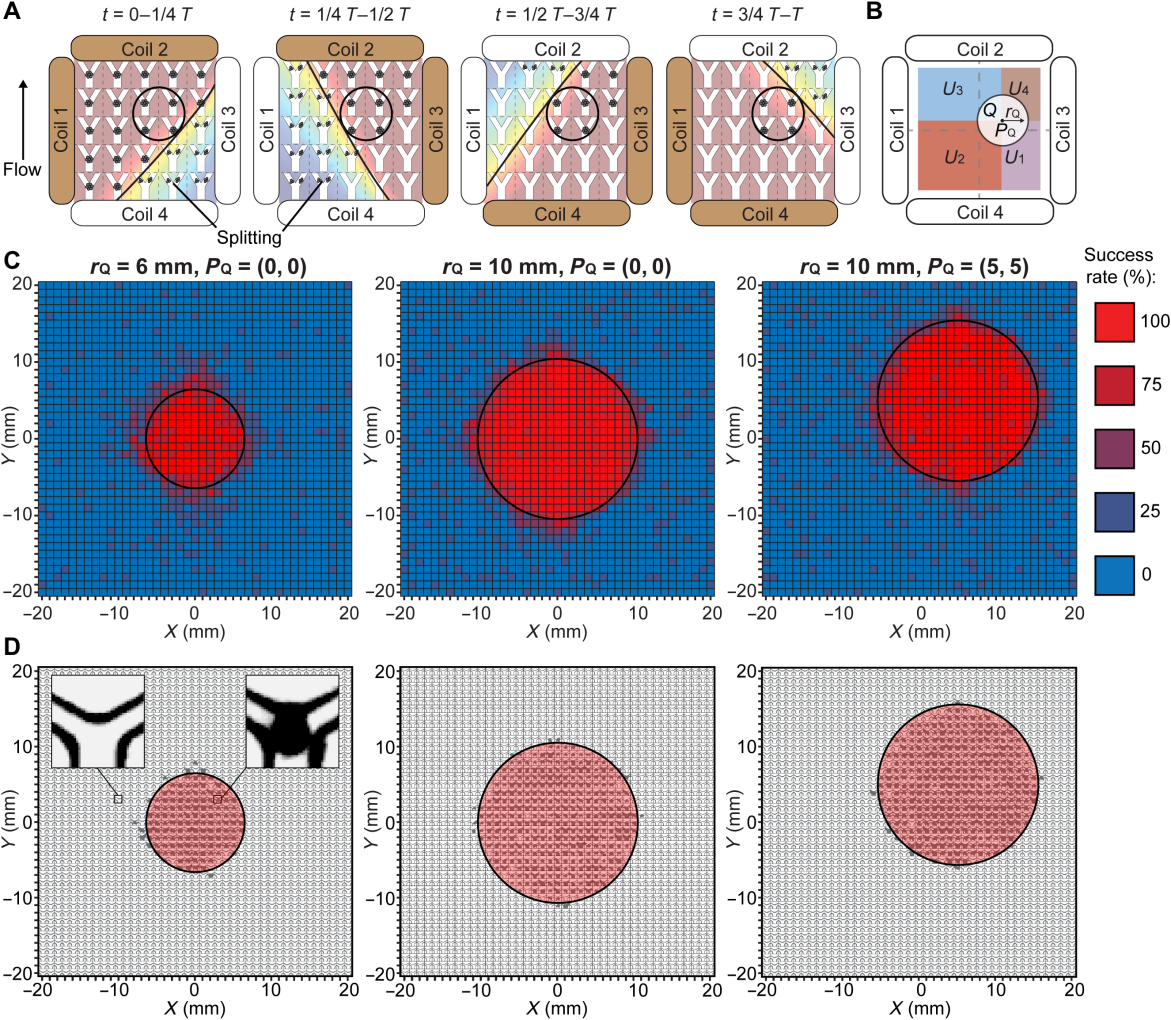
Actuation strategy for selective maintenance of swarm integrity and experimental validation. (**A**) Schematic illustration of the proposed actuation strategy. The black circles indicate the targeted region. The brown and white coils are the dominant and auxiliary coils, respectively. The black lines separate the workspace into regions with the magnetic field strengths higher and lower than *B*_critical_. The black arrowhead denotes the flow direction. (**B**) Schematic illustration of the targeted and nontargeted regions described in the brute-force search. The black circle indicates the targeted region. The radius *r*_Q_ and the center position *P*_Q_ of the targeted region are labeled. The nontargeted subregions *U*_1_, *U*_2_, *U*_3_, and *U*_4_ are highlighted with different colors. (**C**) Experimental success rate of the proposed strategy in maintaining the swarm integrity in three cases. The experimental data in each small square were measured from independent microfluidic channels, and four experiments were repeated to determine the success rate. The black circles indicate the targeted regions. (**D**) Experimental spatial distribution of locations with a success rate of 75% and above in three cases. The left inset shows an empty junction indicating that swarms were split, and the right inset shows a swarm successfully maintained at a junction. The black circles indicate the targeted regions.

When a rotating magnetic field with a strength higher than *B*_critical_ is applied, swarms are stuck at all the junctions covered by the magnetic field. In the case of selective embolization, low magnetic field strength is desired outside a targeted region to degrade the integrity of swarms and prevent unintended blockage. To achieve high selectivity, we developed an actuation strategy to maintain swarm integrity only inside a targeted region. A dynamic magnetic field was created by multiplexing the magnetic fields generated by individual coils over time, as shown in Fig. 3A. From 0 to 1/4*T*, where *T* is an actuation cycle, two adjacent coils serving as dominant coils (brown coils in Fig. 3A) are applied with higher currents to raise the magnetic field strength inside the targeted region. Meanwhile, the other two coils are applied with relatively lower currents and serve as auxiliary coils. The currents applied in the dominant and auxiliary coils are in the opposite directions to attenuate the magnetic field strength outside the targeted region (black circles in Fig. 3A); thus, the splitting of swarms occurs in the attenuated region. From 1/4*T* to 1/2*T*, the dominant and the auxiliary coils are reallocated, as shown in Fig. 3A. Although the magnetic field distribution changes, high magnetic field strength inside the targeted region is ensured. Swarms formed outside the targeted region encounter low-strength magnetic fields; therefore, they cannot maintain their integrity. The current distribution of four coils changes four times to complete a full actuation cycle *T*. Throughout a cycle, the magnetic field strength inside the targeted region is always larger than *B*_critical_, and swarm integrity is maintained at junctions within the targeted region. Outside the targeted region, swarms are periodically subjected to low magnetic field strength and are split by junctions.

To calculate the magnetic field strength generated by an electromagnetic system, the relationship between the current applied in a coil and its resulting magnetic field was investigated. The magnetic field generated by a circular loop exhibits axial symmetry, and the magnetic field strength of an analyzed point can be expressed in the cylindrical coordinate system as (*40*)

$$\left[\begin{array}{c} B_{\rho ,m}\\ B_{\theta ,m}\\ B_{z,m} \end{array}]=[\begin{array}{c} \frac{\mu z_{m}}{2{\pi \rho }_{m}\sqrt{{\left(a+\rho_{m}\right)}^{2}+{z_{m}}^{2}}}(\frac{a^{2}+{z_{m}}^{2}+{\rho_{m}}^{2}}{{z_{m}}^{2}+{\left(\rho_{m}-a\right)}^{2}}E(k_{m})-K(k_{m}))I_{m}\\ 0\\ \frac{\mu }{2\pi \sqrt{{\left(a+\rho_{m}\right)}^{2}+{z_{m}}^{2}}}(\frac{a^{2}-{z_{m}}^{2}-{\rho_{m}}^{2}}{{z_{m}}^{2}+{\left(\rho_{m}-a\right)}^{2}}E(k_{m})+K(k_{m}))I_{m} \end{array}]=[\begin{array}{c} R_{m}I_{m}\\ 0\\ Q_{m}I_{m} \end{array}\right]$$ (7)

where *m* is the index indicating the coils in a system; *B*_ρ*,m*_, *B*_θ*,m*_, and *B_z,m_* are the components of magnetic field strength generated along the unit vectors ${\hat{e}}_{\rho }$, ${\hat{e}}_{\theta }$, and ${\hat{e}}_{z}$, respectively; ρ*_m_*, θ*_m_*, and *z_m_* are the cylindrical coordinates of the analyzed point relative to the coordinate frame of the coil *m* (fig. S4A); *a* is the radius of the coil; μ is the permeability of the coil; *K*(*k*) and *E*(*k*) are the elliptic integrals of the first and second kind, respectively; the argument of the elliptic integrals is ${k_{m}}^{2}=\frac{4\rho_{m}a}{{\left(a+\rho_{m}\right)}^{2}+{z_{m}}^{2}}$; and *I_m_* is the current applied in the coil *m*. These magnetic field strength components are then transformed to the Cartesian coordinate system of the coil *m* as

$$\left[\begin{array}{c} B_{x,m}\\ B_{y,m}\\ B_{z,m} \end{array}]=[\begin{array}{c} B_{\rho ,m}\text{cos}\;\theta_{m}\\ B_{\rho ,m}\text{sin}\;\theta_{m}\\ B_{z,m} \end{array}]=[\begin{array}{c} R_{m}I_{m}\text{cos}\;\theta_{m}\\ R_{m}I_{m}\text{sin}\;\theta_{m}\\ Q_{m}I_{m} \end{array}\right]$$ (8)

where *B_x,m_*, *B_y,m_*, and *B_z,m_* are the components of magnetic field strength along the *x* axis, the *y* axis, and the *z* axis of the coil *m*, respectively. Our electromagnetic system consists of four coils. By transforming the magnetic field strength generated by each coil to the Cartesian coordinate system of our workspace (fig. S4B), the resulting magnetic field strength of a point can be calculated as

$$B_{\left(X,Y,Z\right)}=[\begin{array}{c} B_{X}\\ B_{Y}\\ B_{Z} \end{array}]=[\begin{array}{cccc} Q_{1} & R_{2}\text{cos}\;\theta_{2} & -Q_{3} & -R_{4}\text{cos}\;\theta_{4}\\ R_{1}\text{cos}\;\theta_{1} & -Q_{2} & -R_{3}\text{cos}\;\theta_{3} & Q_{4}\\ R_{1}\text{sin}\;\theta_{1} & R_{2}\text{sin}\;\theta_{2} & R_{3}\text{sin}\;\theta_{3} & R_{4}\text{sin}\;\theta_{4} \end{array}][\begin{array}{c} I_{1}\\ I_{2}\\ I_{3}\\ I_{4} \end{array}]$$ (9)

Our workspace is in the *xz* plane of coils (i.e., θ_1_, θ_2_, θ_3_, and θ_4_ are 0°), as shown in fig. S4. The model in Eq. 9 was validated by experiments. Experimentally measured magnetic field strength was compared to the model-calculated magnetic field strength distribution, as shown in fig. S4C. The percentage difference of magnetic field strength between the experiment and the model was smaller than 10% within the workspace.

To obtain the quantitative current sequences that generate the required dynamic magnetic fields for realizing the actuation strategy, a brute-force search algorithm (*41*) was implemented, which generates and tests all the possible current sequences. The targeted region *Q* was set to be a circular region with its center position *P_Q_* and radius *r_Q_*, as shown in Fig. 3B. The nontargeted region *U* consisted of four subregions that were sequentially exposed to magnetic fields with strengths lower than *B*_critical_ in an actuation cycle *T*. For example, the nontargeted subregions *U*_1_ and *U*_2_ were exposed to low-strength magnetic fields during 0 to 1/4*T* and 1/4*T* to 1/2*T*, respectively. The current sequences must fulfill the following constraints to generate the required dynamic magnetic fields.

1) Currents applied in both dominant coils are sinusoidal, and they have a phase difference of 90° between each other to generate a nonuniform rotating magnetic field

$$\left\{ \begin{array}{c} I_{i,D1}(t)=I\text{sin}(\omega t)\\ I_{i,D2}(t)=I\text{sin}(\omega t+\frac{\pi }{2}) \end{array},t\in T_{i}\right.$$ (10)

where *I*_*i,*D1_ and *I*_*i,*D2_ are the currents applied in the dominant coils, *i* indexes each quarter of the actuation cycle, *I* is the amplitude of the testing current and *I* ϵ [0 A, 5 A] in our experimental setup, ω is the angular frequency, *t* is the time, and *T_i_* indicates the quarter of the cycle. Under this condition, no additional requirement is needed for the currents applied in the auxiliary coils *I*_*i,*A1_ and *I*_*i,*A2_.

2) The magnetic field strength *B* inside the targeted region *Q* is higher than or equal to the calculated *B*_critical_ in every quarter of the cycle.

$$B(t,p,I_{i,D1},I_{i,D2},I_{i,A1},I_{i,A2})\geq B_{\text{critical}},\forall p\in Q,t\in T_{i}$$ (11)

where *p* indicates the point inside the workspace.

3) The magnetic field strength *B* inside the nontargeted subregions *U_i_* is lower than the calculated *B*_critical_ during the corresponding quarter of the cycle

$$B(t,p,I_{i,D1},I_{i,D2},I_{i,A1},I_{i,A2})<B_{\text{critical}},\forall p\in U_{i},t\in T_{i}$$ (12)

The brute-force search outputs a series of current sequences that fulfill the constraints. Using the actuation strategy, no blockage should be formed outside the targeted region. Therefore, we apply the following function to determine the current sequence that generates the weakest magnetic field strength inside the nontargeted region *U* to ensure the swarms are split

$$[\begin{array}{cccc} I_{1} & I_{2} & I_{3} & I_{4} \end{array}]=\underset{I_{1},I_{2},I_{3},I_{4}}{\text{arg min}}\sum_{t=0}^{T}\sum_{p=1}^{U}B(t,p,I_{1},I_{2},I_{3},I_{4}),\forall p\in U,t\in T$$ (13)

where *I*_1_, *I*_2_, *I*_3_, and *I*_4_ are the current sequences applied in coils 1, 2, 3, and 4, respectively.

The proposed actuation strategy was validated by experiments. Microfluidic channels with 120° branching angles were placed 1 mm apart from each other inside the workspace, as shown in fig. S5. Magnetic particles suspended in PBS solution were injected into the channels individually at a flow rate of 80 μm/s. From the model in Eq. 6, *B*_critical_ was calculated to be 16 mT. Three targeted regions were set sequentially, (i) *P*_Q1_ = (0 mm, 0 mm), *r*_Q1_ = 6 mm; (ii) *P*_Q2_ = (0 mm, 0 mm), *r*_Q2_ = 10 mm; and (iii) *P*_Q3_ = (5 mm, 5 mm), *r*_Q3_ = 10 mm. Three corresponding current sequences were obtained from the brute-force search, and then three different dynamic magnetic fields were generated. The simulation results of the dynamic magnetic fields are summarized in figs. S6 and S7 and movie S3. Figure 3C shows the success rate of maintaining swarm integrity at different locations (each square represents a location) inside the workspace, under the actuation of dynamic magnetic fields. At each location, experiments were repeated four times to determine the success rate (e.g., a 50% success rate means successfully maintaining swarm integrity two times out of four trials). Experimental results showed that the average success rates were 91 and 6% inside and outside the targeted region, respectively, as summarized in Fig. 3C. Figure 3D shows the experimental spatial distribution of locations with the success rate of 75% and above. We further used the Jaccard index (*42*) *I*_JAC_ to evaluate the selectivity of the proposed strategy

$$I_{\text{JAC}}=\frac{T_{s}}{T_{s}+T_{\text{NS}}+N_{s}}$$ (14)

where *T*_s_ is the number of targeted junctions with swarms, *T*_NS_ is the number of targeted junctions without swarms, and *N*_s_ is the number of nontargeted junctions with swarms. On the basis of the results in Fig. 3D, the average index was determined to be 0.9, indicating a high selectivity of the proposed strategy.

### Embolization in microfluidic channels

**Fig. 4.**
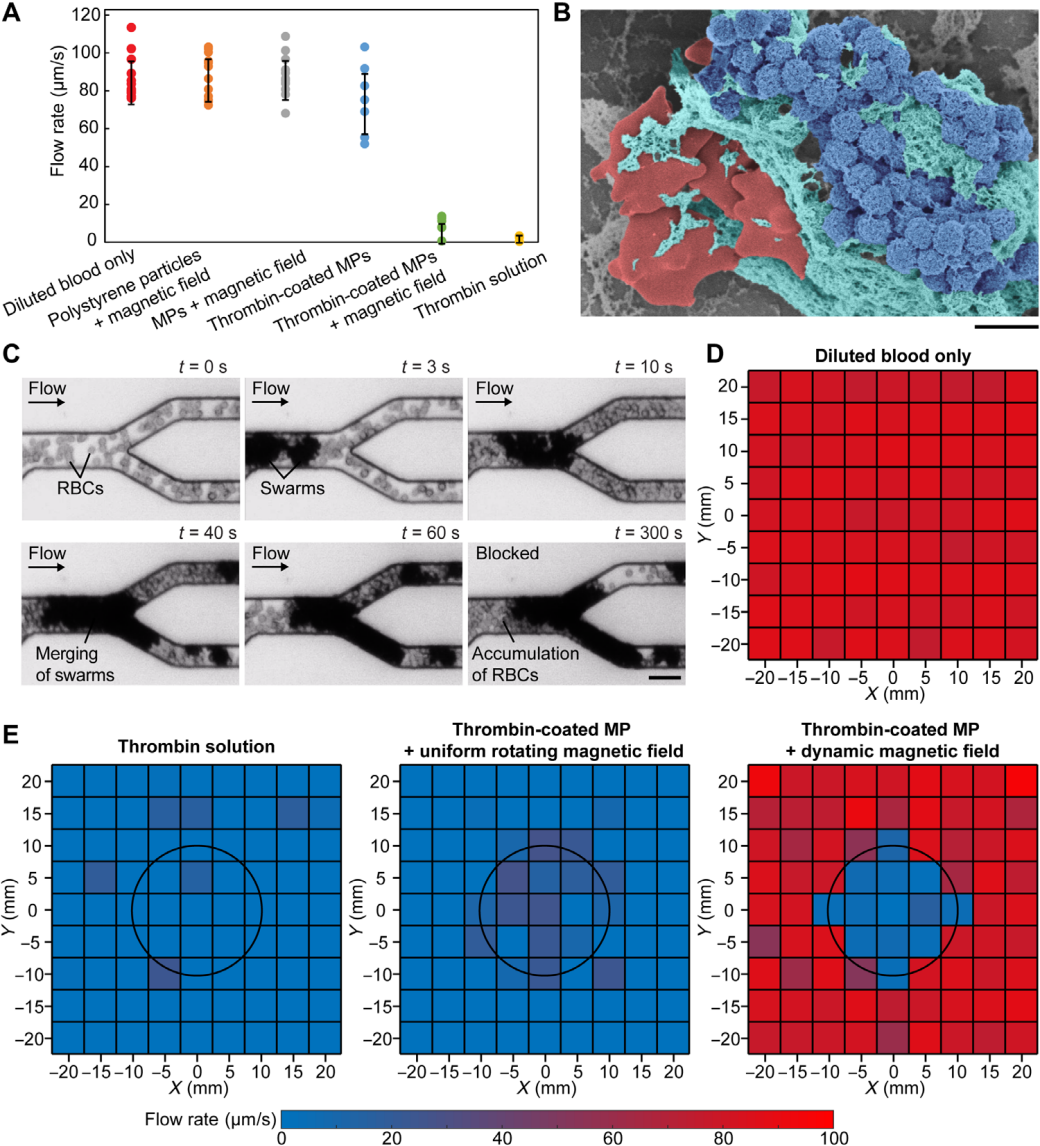
Embolization in microfluidic channels. (**A**) Different conditions for reducing blood flow rate. The flow rates were measured when the conditions were kept activated for 10 min. The error bars represent the SD of 10 trials. MPs denote magnetic particles. (**B**) Scanning electron microscopy image of a clotting swarm. For visualization, porcine RBCs, fibrin meshes, and magnetic particles were artificially colored in red, green, and blue, respectively. Scale bar, 2 μm. (**C**) Experimental results of embolization in microchannels using thrombin-coated magnetic particles. Scale bar, 20 μm. (**D**) The input flow rate of diluted porcine blood in the microfluidic channels (average flow rate: 83 μm/s). (**E**) Experimentally measured flow rate in the microfluidic channels under different embolization conditions. The flow rates were measured when the conditions were kept activated for 10 min. For (D) and (E), the data in each small square were measured from independent microfluidic channels, and three experiments were conducted to obtain an average flow rate. The black circles indicate the targeted region.

To test the effectiveness of using magnetic particle swarms for blocking blood flow, blood flow rate was measured under different conditions and compared in Fig. 4A. To ensure visibility under optical microscopy, porcine whole blood was diluted and then injected into microfluidic channels with 120° branching angles (see Materials and Methods). The flow rate was quantified by measuring the speed of the RBCs, and 10 measurements were made to determine the average flow rate. The injected flow rate was maintained at an average of 84 μm/s (red data points in Fig. 4A). Correspondingly, *B*_critical_ was calculated to be 16.6 mT from the model in Eq. 6.

Polystyrene particle suspension (orange data points in Fig. 4A) and magnetic particle suspension (gray data points in Fig. 4A) were injected into the microfluidic channels, respectively, and a uniform rotating magnetic field with a strength of 20 mT was applied to maintain swarm integrity. It was found that the average flow rate was not reduced and remained unchanged at 84 μm/s for both the polystyrene particle suspension and the magnetic particle suspension. Since polystyrene particles do not respond to magnetic fields, they moved with the flow without causing blockage. For magnetic particles, they formed swarms at the junctions; however, there still existed space between particles inside the swarm, through which the deformable RBCs passed, and the blood flow rate was not greatly reduced.

Using magnetic particles coated with thrombin, when no magnetic field was applied, the average flow rate was reduced slightly to 72 μm/s (blue data points in Fig. 4A), which may be due to the formation of small clusters that influenced the blood flow. When a 20-mT uniform rotating magnetic field was applied, the average flow rate was greatly reduced to 4 μm/s (green data points in Fig. 4A) because thrombin-coated magnetic particles captured RBCs to fill the interspace and formed clotting swarms that blocked the flow. The scanning electron microscopy image in Fig. 4B shows magnetic particles (blue) and RBCs (red) trapped by fibrin meshes (green) to form a clotting swarm. The experimental results using thrombin-coated magnetic particles to occlude a branched channel are shown in Fig. 4C and movie S4. Last, when thrombin solution was directly injected into the microfluidic channels, the average blood flow rate was greatly reduced to 1 μm/s (yellow data points in Fig. 4A) because fibrinogen in the blood formed clots as anticipated. Compared to the passive embolization induced by thrombin solution, the blockage of channels using thrombin-coated magnetic particles were actively triggered on demand by applying a rotating magnetic field with a strength higher than *B*_critical_.

We then validated that our proposed strategy was able to actuate thrombin-coated magnetic particles for selective embolization. We also tested the effectiveness of using thrombin solution in selective embolization. Microfluidic channels with 120° branching angles were placed 5 mm apart from each other inside the workspace. We injected diluted porcine blood into the microfluidic channels individually, and the flow rate was maintained at an average of 83 μm/s, as shown in Fig. 4D. The blood flow rate under different embolization conditions is summarized in Fig. 4E. The targeted region was set to be 10 mm in radius and centered at (0 mm, 0 mm), as indicated by the black circles in Fig. 4E. At each location, experiments were repeated three times to determine the average flow rate. When thrombin solution was injected, the average flow rate was greatly reduced to 2 μm/s both inside and outside the targeted region, confirming that thrombin solution caused embolization nonselectively. Similarly, when thrombin-coated magnetic particles were actuated with a 20-mT uniform rotating magnetic field, the average flow rate was reduced to 4 μm/s inside the entire workspace because clotting swarms were formed at all the junctions covered by the uniform rotating magnetic field. From the brute-force search, the current sequence that fulfilled Eqs. 10 to 13 was obtained, and the corresponding dynamic magnetic field was generated. The average flow rate inside the targeted region was reduced to 6 μm/s, representing a 93% reduction of flow rate, while the flow rate outside the targeted region was not notably influenced (~79 μm/s). These results demonstrated that our actuation strategy together with thrombin-coated magnetic particles was able to achieve selective embolization with minimal unintended blockage outside a targeted region.

### Embolization in porcine organs

**Fig. 5.**
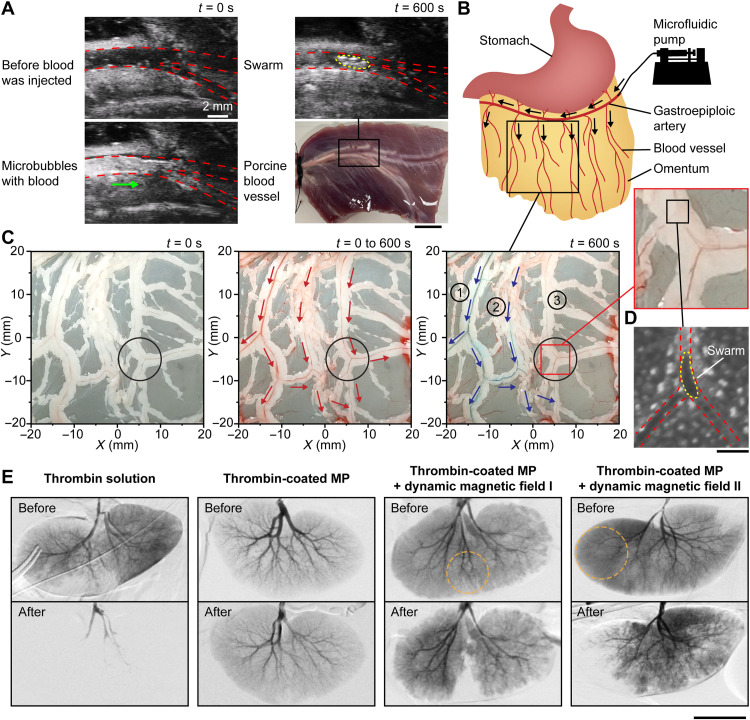
Embolization in porcine organs. (**A**) Formation of a clotting swarm at the junction of an ex vivo porcine blood vessel. The red dashed lines outline the blood vessel and junction, and the yellow dashed line outlines the clotting swarm. The green arrow shows the flow direction of microbubbles. Scale bar, 10 mm. (**B**) Schematic illustrating the injection site of an ex vivo porcine omentum in experiments. Black arrows indicate the flow direction. (**C**) Selective embolization in the blood vessel network of an ex vivo porcine omentum with the targeted region centered at (5 mm, −5 mm). The black circles indicate the targeted region, the red arrows indicate the blood flow direction, and the blue arrows indicate the flow direction of blue dye. (**D**) Optical microscopy image showing a swarm formed at the targeted junction of an ex vivo porcine omentum. The red dashed lines outline the blood vessel and junction, and the yellow dashed lines outline the magnetic particle swarm. Scale bar, 200 μm. (**E**) Digital subtraction angiography results of in vivo porcine kidneys under different embolization conditions. The orange dotted circles indicate the targeted regions. Scale bar, 50 mm.

To verify the effectiveness of using magnetic particle swarms for blocking blood flow in tissues, we conducted experiments in a porcine blood vessel ex vivo. Blocking a porcine blood vessel using microrobotic swarms is shown in Fig. 5A. The blood vessel with a branching angle of 30° was imaged by an ultrasound imaging system (Vevo 3100, FUJIFILM VisualSonics). When microbubbles (i.e., ultrasound imaging contrasts) were injected with blood, they moved freely with blood and brightened up the blood vessel and junction due to their high echogenicity, indicating that the blood vessel was unblocked. Thrombin-coated magnetic particles were then injected into the blood vessel at a flow rate of 80 μm/s. The corresponding *B*_critical_ was calculated to be 23 mT using the model in Eq. 6, and a 25-mT uniform rotating magnetic field was sustained for 10 min to form swarms at the junction. The brightened-up spot (outlined by a yellow dashed line) at the junction indicated the formation of a swarm [i.e., the contrast was enhanced by the swarm (*29*)], and the microbubbles stopped moving, confirming the blockage of the blood vessel by the swarm.

Furthermore, we tested the proposed actuation strategy for selective embolization in the blood vessel network of an ex vivo porcine omentum, as shown in Fig. 5 (B and C) and movie S5. The greater omentum is a highly vascularized peritoneal fold and a site for tumor formation and metastasis (*43*). Thrombin-coated magnetic particles were injected with porcine blood into the greater omentum from the gastroepiploic artery, as illustrated in Fig. 5B, at a flow rate of 80 μm/s. The targeted region was set to be 6 mm in radius and centered at (5 mm, −5 mm). The branching angle of the targeted vessel was approximately 100° and *B*_critical_ was calculated to be 20 mT. A current sequence was obtained from the brute-force search, and the corresponding dynamic magnetic field was sustained for 10 min. Then, blue dye was injected from the same site (i.e., the gastroepiploic artery). The blood vessels outside the targeted region (blood vessels 1 and 2) were colored, while the blood vessel associated with the targeted junctions (blood vessel 3) remained red/unaffected, as shown in Fig. 5C. When the targeted junctions were blocked, the fluidic resistance of the associated blood vessel 3 became substantially higher than that of blood vessels 1 and 2 outside the targeted region; thus, blue dye bypassed blood vessel 3. Figure 5D shows the swarm observed using optical microscopy at the targeted junction, which blocked the vessel.

We then tested the proposed actuation strategy for selective embolization in in vivo porcine kidneys, as shown in Fig. 5E. Before embolization, blood vessels and kidneys appeared darker than backgrounds when iohexol [i.e., an imaging contrast for digital subtraction angiography (DSA)] was injected into renal arteries. The kidneys were then embolized under different conditions for 10 min, after which iohexol was injected again to investigate the changes in the blood vessels and kidneys. Thrombin solution was supplied to the first kidney, and after embolization, the kidney and most of the blood vessels did not appear under DSA imaging, indicating that thrombin solution caused blockage without selectivity. Thrombin-coated magnetic particle solution was supplied to the second kidney, with no magnetic fields applied, and after 10 min, no change was observed in the blood vessels and kidney, indicating that thrombin-coated magnetic particles alone did not block the vessels. The third and the fourth kidneys were injected with thrombin-coated magnetic particle solution and were exposed to dynamic magnetic fields that corresponded to the targeted regions with 40 and 50 mm diameter, respectively (outlined by orange dotted circles). After 10 min, the targeted regions of the third kidney (middle portion) and the fourth kidney (left portion) did not appear under DSA imaging, while the other regions appeared darker, demonstrating that the proposed strategy was able to realize in vivo selective embolization.

## DISCUSSION

An actuation strategy was developed for maintaining swarm integrity at junctions inside a targeted region to block the blood flow and realize selective embolization. We revealed the relationships between branching angle, fluid viscosity, flow rate, magnetic field strength, and swarm integrity, via analytical modeling. On the basis of the model, an actuation strategy was developed, and a brute-force search algorithm was implemented to obtain current sequences that generate required dynamic magnetic fields. Using thrombin-coated magnetic particles, we validated the proposed strategy in microfluidic channels. Experimental results showed that selective embolization was achieved with minimal unintended blockage outside the targeted region. Moreover, the proposed strategy realized in vivo selective embolization in porcine kidneys.

The developed actuation strategy enables magnetic particle swarms to simultaneously embolize the blood vessels inside a targeted region by selectively maintaining their integrity. Although navigated locomotion of microrobotic swarms has been shown (*7*–*9*, *23*–*26*, *44*–*48*), independent locomotion control of multiple homogeneous swarms is challenging because they exhibit identical behaviors in the same actuation field. Consequently, when the tasks involve multiple scattered blood vessels, the swarms need to be navigated to perform tasks in each blood vessel one after another, resulting in prolonged surgical time. Moreover, in vivo navigation of swarms remains challenging because effective in vivo imaging of swarms is still under investigation (*46*–*50*). Without the need of swarm navigation, our method uses blood flows to distribute swarms and blood vessel junctions to constrain the motion of swarms, and we designed dynamic magnetic fields to maintain the swarm integrity inside a targeted region while breaking the swarm integrity outside it.

Other than embolization, the high selectivity of the developed actuation strategy would enable new capabilities for biomedical applications such as hyperthermia. Using the strategy to explore targeted hyperthermia, magnetic agents inside the targeted region are driven by high magnetic field strengths, while agents outside it experience attenuated magnetic field strength, i.e., agents inside the targeted region would deliver more heat over time than those outside it, which would enhance the effect of local hyperthermia and reduce damage to surrounding healthy tissues. The strategy may also provide design solutions for complex dynamic magnetic field–driven micromanipulation; e.g., magnetic agents inside the targeted region gain more rotational energy to perform micromanipulation tasks selectively.

This study shows that magnetic particle swarms can be actuated to achieve selective embolization. Microrobotic swarms driven by the developed actuation strategy can provide a potential solution for selective embolization to mitigate complications [e.g., stroke and blindness (*31*, *32*, *34*)] caused by the present passive, nonselective embolization technique.

## MATERIALS AND METHODS

### Materials and preparation for experiments

Superparamagnetic particles (1 μm diameter, Dynal Biotech) were suspended in PBS (Gibco) solution to obtain magnetic particle suspension with a concentration of 2 × 10^9^ particles/ml. The superparamagnetic particles were coated with thrombin (from bovine plasma, Sigma-Aldrich) to obtain thrombin-coated magnetic particles (*51*). Briefly, 1 ml of particles (2 × 10^9^ particles/ml in pH 9.4 carbonate buffer) was combined with 3 ml of bovine plasma thrombin (1 U/ml) and then incubated for 24 hours at 4°C. After incubation, the thrombin-coated magnetic particles were washed and reconstituted with PBS solution. Other than 1 U/ml thrombin, magnetic particles were also tested with 2, 4, 6, and 8 U/ml thrombin solution, and their effectiveness in flow rate reduction was characterized in fig. S8. Magnetic particles coated with thrombin solution (8 U/ml) were used in the experiments. To warrant the formation of clotting swarms, a sufficiently long sustaining time of 10 min was set. Thrombin solution (8 U/ml) was also used in the experiments. Polystyrene particles (5 μm in diameter, Sigma Aldrich) were suspended in PBS solution to obtain polystyrene particle suspension. Porcine whole blood (Innovative Research) was diluted using PBS solution with a ratio of 1:10, and fibrinogen with a concentration of 10 μg/ml was added into the diluted blood in a ratio of 1:1 to maintain a similar level of concentration with whole blood (*52*). Microfluidic channels were made of polydimethylsiloxane, and the design is shown in fig. S5. Magnetic actuation was conducted in a magnetic system, as shown in fig. S9. The system consisted of four identical magnetic coils, a camera, and a syringe pump (Harvard Apparatus Pump 11). The actuation signals were generated by a computer, and then the current sequences were supplied to the coils to generate dynamic magnetic fields inside the workspace. Magnetic field strengths were measured by a gaussmeter (Model 410, Lake Shore Cryotronics).

### Generation of magnetic particle swarms

Low Reynolds number (*Re*) was estimated in our case (*Re* ≈ 10^−5^). To determine the driving frequency of a dynamic magnetic field where disk-like swarms are formed, a modified Mason number *R*_T_ was used and expressed as (*53*)

$$R_{T}=16\frac{\mu_{0}\eta \omega }{{\chi_{p}}^{2}B^{2}}\frac{N^{3}}{\left(N-1)(\text{ln}(N/2)+2.4/N\right)}$$ (15)

where η is the viscosity of fluid, ω is the angular frequency of the rotating field, χ_p_ is the effective magnetic susceptibility of the particle, μ_0_ is the permeability of free space, *B* is the magnetic field strength, and *N* is the number of particles in the chain. Particle chains rotate as a rigid rod if the modified Mason number *R*_T_ is smaller than unity. Otherwise, fragmentation of particle chains occurs (*53*). With the modified Mason number of 1, the number of particles of 20, the magnetic field strength of 20 mT, the effective magnetic susceptibility of particle of 0.3, and the fluid viscosity of 1 mPa·s, the transformation of chains into swarms was estimated to occur at ≈2 Hz. In our case, particle chains showed signs of fragmentation into swarms when the field frequency was 5 Hz, with a magnetic field strength of 20 mT (fig. S1). When the field frequency was increased to 15 Hz, 95% of the chains turned into swarms.

### Influence of van der Waals and electrostatic forces on swarm integrity

Other than magnetic dipole-dipole interactions, magnetic particles also interact with each other and the wall through van der Waals **F**_**vdw**_ and electrostatic forces **F**_**el**_ (*54*). The van der Waals and electrostatic forces can be expressed as (*55*)

$$F_{\text{vdw}}=-(\nabla W_{\text{vdw}})=-\frac{A_{h}R}{6h^{2}}$$ (16)

$$F_{\text{el}}=-(\nabla W_{\text{el}})=64{\kappa \pi \varepsilon }_{0}\varepsilon_{p}R{\left(\frac{k_{B}T}{e}\right)}^{2}{\text{tanh}}^{2}(\frac{e\varphi_{p}}{4k_{B}T})\text{exp}(-\kappa h)$$ (17)

$$\kappa =\sqrt{\frac{2N_{A}e^{2}I}{\varepsilon_{0}\varepsilon_{p}k_{B}T}}$$ (18)

where *W*_vwd_ is the van der Waals potential, *W*_el_ is the electrostatic interaction energy, *A*_h_ is the Hamaker constant, *h* is the distance between the particles or that between the wall and particles, κ^−1^ is the Debye length, ε_0_ is the permittivity of vacuum, ε_p_ is the relative permittivity of blood, *k*_B_ is the Boltzmann constant, *T* is the temperature, *e* is the elementary electric charge, φ_p_ is the surface potential of the particles, *N*_A_ is the Avogadro number, and *I* is the ionic strength of the solution. The coefficient *R* is *a*_p_/2 for the case between two spherical particles with the same diameter and *a*_p_ for the case between a wall and a spherical particle, where *a*_p_ is the radius of the particles. In blood, the attractive van der Waals and repulsive electrostatic forces between two particles are ≈8.3 × 10^−13^ and ≈5.4 × 10^−63^ N, respectively, while that between a wall and a particle are ≈1.7 × 10^−12^ and ≈1.1 × 10^−62^ N, respectively (data in table S1). Compared to the magnetic dipole-dipole interaction force within a particle swarm (≈10 × 10^−12^ N in a magnetic field strength of 10 mT), the attractive van der Waals interaction between the wall and particles potentially affects swarm integrity. To compensate for the uncertain effects, a model calibration factor δ is introduced into Eq. 6. The model calibration factor δ is calculated as

δ=arg minδ∑0vf∑π6γ∣δBcritical(vf,γ)−Bexp∣ (19)

where **v**_**f**_ is the flow rate, γ is the branching angle of the junction, and *B*_exp_ is the measured magnetic field strength.

### Numerical calculation of critical magnetic field strength *B*_critical_

The model in Eq. 6 was used to calculate an average critical magnetic field strength *B*_critical_ with the following parameters: 1 μm for the diameter of the magnetic particles, 0.58 for the effective magnetic susceptibility of the particles, 0.35 for the drag coefficient correction factor of a spherical particle within a compacted swarm (*36*), and 1.4 mPa·s (*39*) and 1 mPa·s for the viscosity of porcine blood plasma and PBS solution, respectively. As shown in Fig. 1B, the angles of the magnetic dipole-dipole interaction force vectors (exerted on the tip-particle by the neighboring particles) with respect to the *x* axis are $\theta_{1}\approx \frac{\pi }{6}-\frac{\gamma }{3}$, $\theta_{2}\approx \frac{\pi }{6}-\frac{\gamma }{3}$, and $\theta_{3}\approx -\frac{\pi }{2}$, where γ is the branching angle. The distances between the centers of the neighboring particles and that of the tip-particle are *r*_1_ ≈ *d*, *r*_2_ ≈ *d*, and $r_{3}\approx 2d\text{sin}(\frac{\pi }{6}+\frac{\gamma }{3})$. The range of flow rate was 0 to 120 μm/s, and branching angles of 30^°^, 60^°^, 90^°^, and 120^°^ were used. The model calibration factor was calculated to be 3.6 based on the experimental results in Fig. 2 (A and B).

### Generality of model

The generality of the model in Eq. 6 was investigated by comparing its predictions to those of three 3D swarm integrity models (fig. S3). Representative disk-like swarms and ellipsoid-like swarms were obtained from COMSOL simulation (labeled by green boxes in fig. S3A) in which a rotating magnetic field with a frequency of 10 Hz and a field strength of 10 mT was applied in the *xy* plane. On the basis of the simulation results, the tip-particle configurations of 3D swarms at junctions were analyzed (labeled by black dotted circles in fig. S3A), and three 3D swarm integrity models were developed according to

$$B_{\text{critical}}={\left(3\pi \eta \text{dk}(v_{p}-v_{f}){\left([\begin{array}{ccc} c_{\text{int},1} & \cdots & c_{\text{int},n} \end{array}][\begin{array}{c} \text{cos}\;\alpha_{1}\text{cos}\;\beta_{1}\\ \vdots \\ \text{cos}\;\alpha_{n}\text{cos}\;\beta_{n} \end{array}]-\text{tan}\frac{\gamma }{2}[\begin{array}{ccc} c_{\text{int},1} & \ldots & c_{\mathrm{int},n} \end{array}][\begin{array}{c} \text{cos}\;\alpha_{1}\text{sin}\;\beta_{1}\\ \vdots \\ \text{cos}\;\alpha_{n}\text{sin}\;\beta_{n} \end{array}]\right)}^{-1}\right)}^{\frac{1}{2}}\delta$$ (20)

where η is the dynamic viscosity of the fluid, *d* is the diameter of the particle, *k* is a drag coefficient correction factor, **v**_**f**_ is the flow rate, **v**_**p**_ is the velocity of the tip-particle, *n* is the total number of the nearest particle surrounding tip-particle *j*, γ is the branching angle of the junction, α is the angle between the magnetic dipole-dipole interaction force vector **F**_**int,ij**_ and the *xy* plane, β is the angle of the projection of vector **F**_**int,ij**_ in the *xy* plane with respect to the *x* axis, and δ is the model calibration factor in Eq. 16. The coefficient can be expressed as $c_{\text{int,ij}}=\frac{3V^{2}{\chi_{p}}^{2}}{4{\pi \mu }_{0}{r_{\text{ij}}}^{4}}[(1-5{\left(\hat{B}\cdot {\hat{r}}_{\text{ij}}\right)}^{2}){\hat{r}}_{\text{ij}}+2(\hat{B}\cdot {\hat{r}}_{\text{ij}})\hat{B}]$, where *V* is the volume of the particle, *r_ij_* and ${\hat{r}}_{\text{ij}}$ are the distance and unit vector between the center of particle *i* and that of tip-particle *j*, respectively, χ_p_ is the effective magnetic susceptibility of the particle, μ_0_ is the permeability of free space, and $\hat{B}$ is the unit vector of the magnetic field.

The parameters of the 3D swarm integrity models are included in note S1, and their predictions are shown in fig. S3 (B and C). Statistical *F* tests were conducted to evaluate the differences between the variance of the 2D swarm integrity model and that of each 3D swarm integrity model. The statistical significance in each comparison was evaluated as *P* < 0.05 for a significance level with a null hypothesis of no difference between the variances. The *P* value of each comparison was larger than 0.05, as listed in table S2, indicating that the model predictions are not significantly different. The relative errors of each model are also summarized in table S3.

### Numerical calculation of actuation current sequences

In the brute-force search calculation, the period of a full actuation cycle *T* was determined to be 12 s because 3 s was sufficient for the splitting of the swarms in experiments.

### In vivo experiment setup

The studies were conducted in Silver Snake Clinical Center (Guangzhou, China), and the animal handling procedures were approved by Silver Snake Clinical Center Institutional Animal Care and Use Committee (SS-2022-GDSZ 1). Two 8- to 9-week-old female Xizang pigs, weighing 26 and 30 kg, respectively, were studied. Anesthesia was maintained during the entire process. The kidneys were randomly divided into four groups, and each group of the kidneys was embolized under different conditions. The magnetic field strength that can be generated by our in vivo coil system was up to 30 mT, which can maintain swarm integrity at a blood flow rate of ~100 μm/s. Before embolization, the blood flow rate inside the kidney was temporarily adjusted to 100 μm/s. The blood flow adjustment was achieved by using a surgical clamp, which is a common practice in surgery (*56*). The blood vessel networks and blood flow inside the pig kidneys were detected by DSA imaging (CGO-2100, Wandong). Each kidney was then treated with its corresponding embolization condition. Thrombin solution (50 U/ml) and magnetic particles coated with thrombin solution (8 U/ml) were used. The concentration of thrombin-coated magnetic particle solution was 10 mg/ml. Dynamic magnetic fields corresponding to different targeted regions (40 and 50 mm diameter, respectively) were applied. After embolization, the surgical clamp was removed to restore normal blood flow. Then, ioxehol was injected through a catheter for DSA imaging of the blood vessel networks of the kidneys. The experiment setup is shown in fig. S10.

## Supplementary Materials

### This PDF file includes:

Figs. S1 to S10 Tables S1 to S3 Note S1
