# Supplementary material for: Microrobotic swarms for selective embolization
Source: Sci Adv. 2022 Jul 20;8(29):eabm5752. doi: 10.1126/sciadv.abm5752 (PMC9299543; doi:10.1126/sciadv.abm5752)
Supplement: Supplementary file 1 — Figs. S1 to S10 Tables S1 to S3 Note S1 [file sciadv.abm5752_sm.pdf]

Science Advances
AAAS

Supplementary Materials for

Microrobotic swarms for selective embolization

Junhui Law *et al.*

Corresponding author: Peng Song, songpeng2021@vip.sina.com; Jiangfan Yu, yujiangfan@cuhk.edu.cn;
Yu Sun, yu.sun@utoronto.ca

Sci. Adv. **8**, eabm5752 (2022)
DOI: 10.1126/sciadv.abm5752

The PDF file includes:

Figs. S1 to S10
Tables S1 to S3
Note S1
Legends for movies S1 to S5

Other Supplementary Material for this manuscript includes the following:

Movies S1 to S5

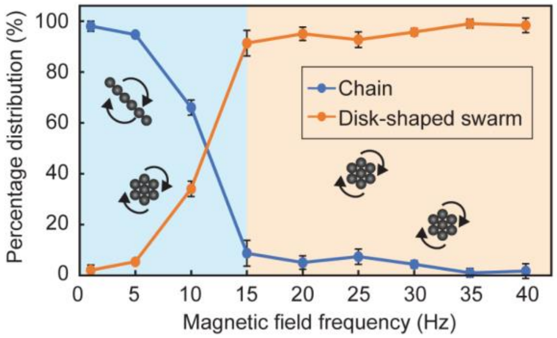

Fig. S1. Swarm patterns in rotating magnetic fields with different frequencies. Percentage distribution of particle chains and disk-shaped swarms inside the workspace when rotating magnetic fields with different frequencies were applied. In our case, particle chains showed signs of fragmentation into swarms when the driving frequency was 5 Hz. When the frequency was further increased to 15 Hz, 95% of the chains turned into swarms. The magnetic field strength applied was 20 mT. Each data point represents the average of three experiments. The error bars represent standard deviation.

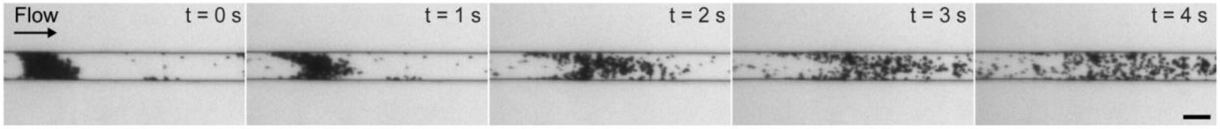

Fig. S2. Degradation of swarm integrity outside a magnetic field. Disassembly of a swarm by flow outside a magnetic field region. Scale bar, 10 µm.

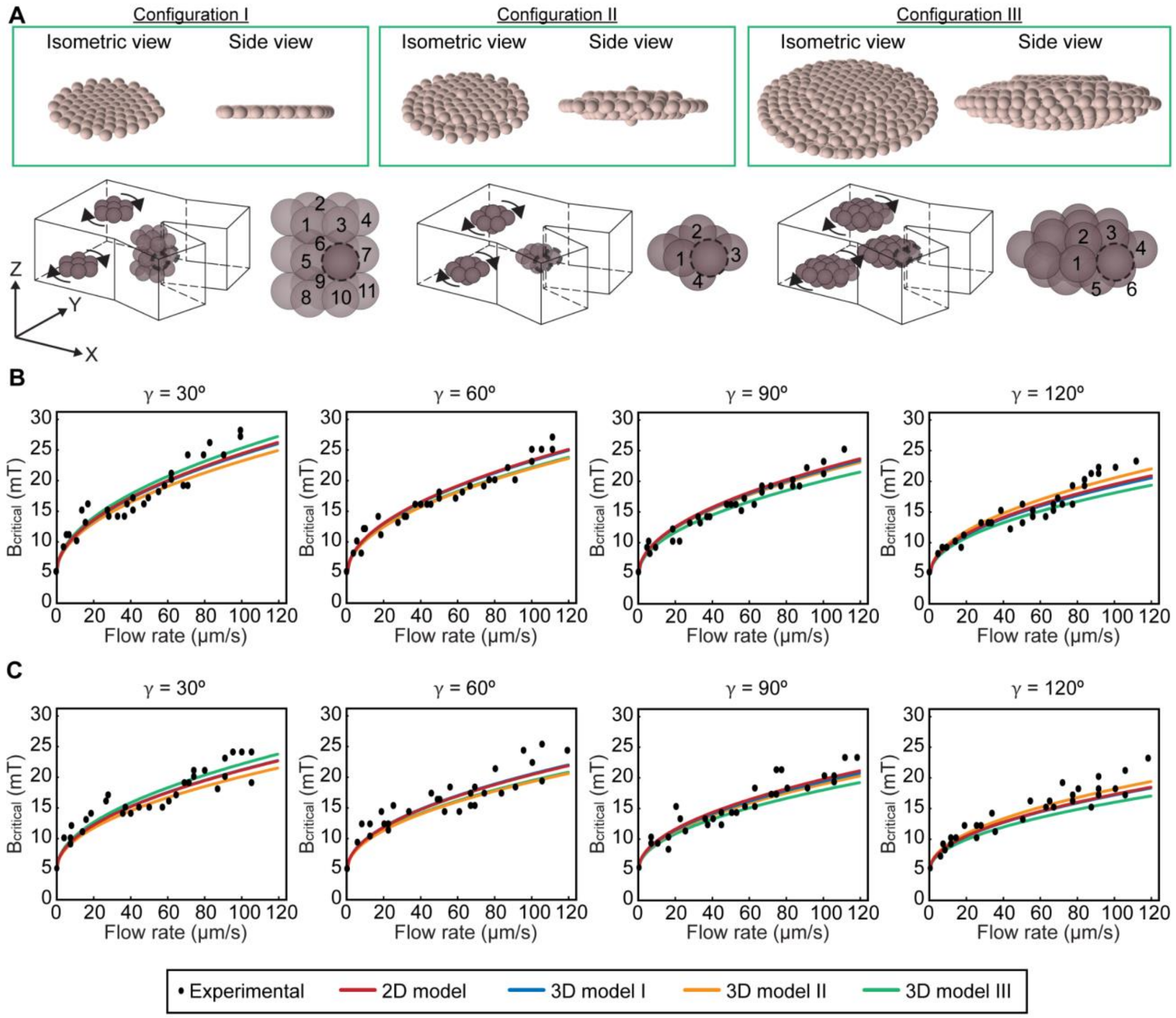

Fig. S3. Three-dimensional swarm integrity models. (**A**) Schematics illustrating the configurations of particle swarms at junctions. The swarms I, II, and III in the green boxes are simulated by using COMSOL. The black dotted circles indicate tip-particles, and the numbers index the neighboring particles. (**B** and **C**) The relationship between critical magnetic field strength $B_{critical}$ and flow rate at junctions with different branching angles γ in porcine whole blood and PBS solution, respectively. The red, blue, orange, and green lines are the $B_{critical}$ calculated by using the two-dimensional swarm model (2D model), and three-dimensional swarm models based on configuration I (3D model I), configuration II (3D model II), and configuration III (3D model III), respectively.

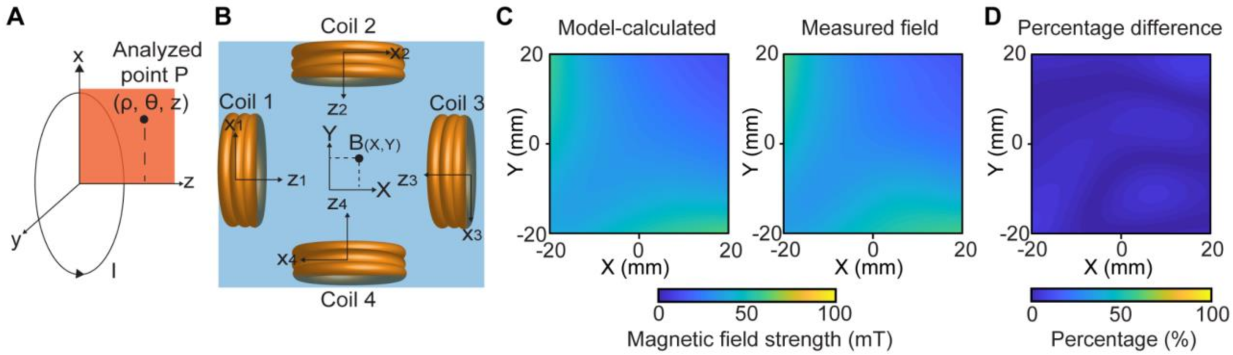

Fig. S4. Calculation of magnetic field strength. (**A**) Schematic of a circular current loop in cylindrical coordinate. The black dot indicates the analyzed point. The black arrow indicates the current direction. (**B**) The coordinate frame of each coil in the frame of the workspace. The workspace is in the x-z plane of the coils (blue rectangle). (**C**) The magnetic field strength distribution calculated by the model in Eq. 9 and the experimentally measured field strength. (**D**) The percentage difference between the model-calculated and the experimental measurements inside the workspace. In (C) and (D), coils 1 and 2 were supplied with 2 A.

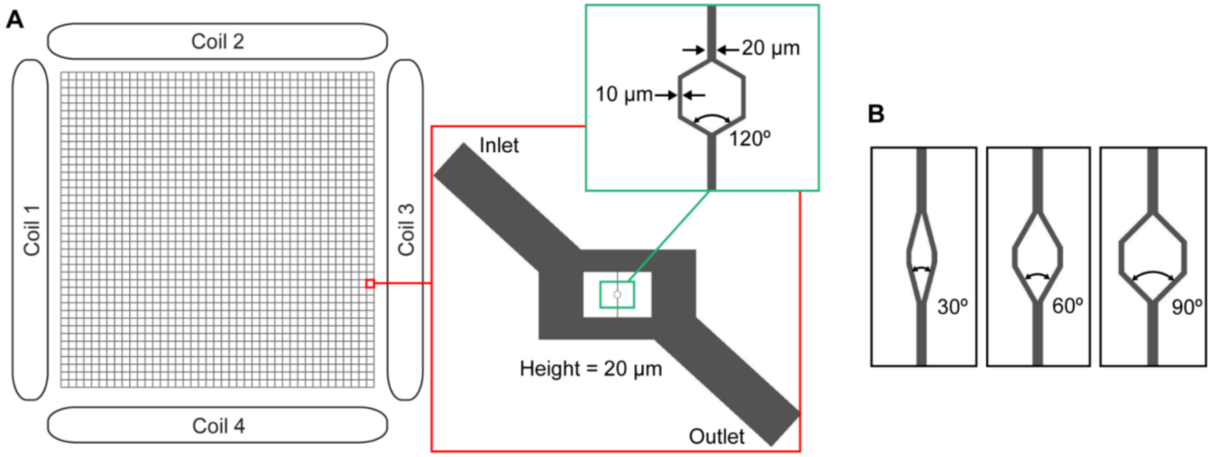

Fig. S5. Experimental setup of microfluidic channels. (**A**) Experimental setup of microfluidic channels inside the workspace. A microfluidic device (in the red box) was placed at the center of each small square. Each microfluidic device consisted of a branched channel (in the green box). (**B**) Channels with different branching angles. The width of the large and small branch is 20 μm and 10 μm, respectively, as shown in the green box of (A).

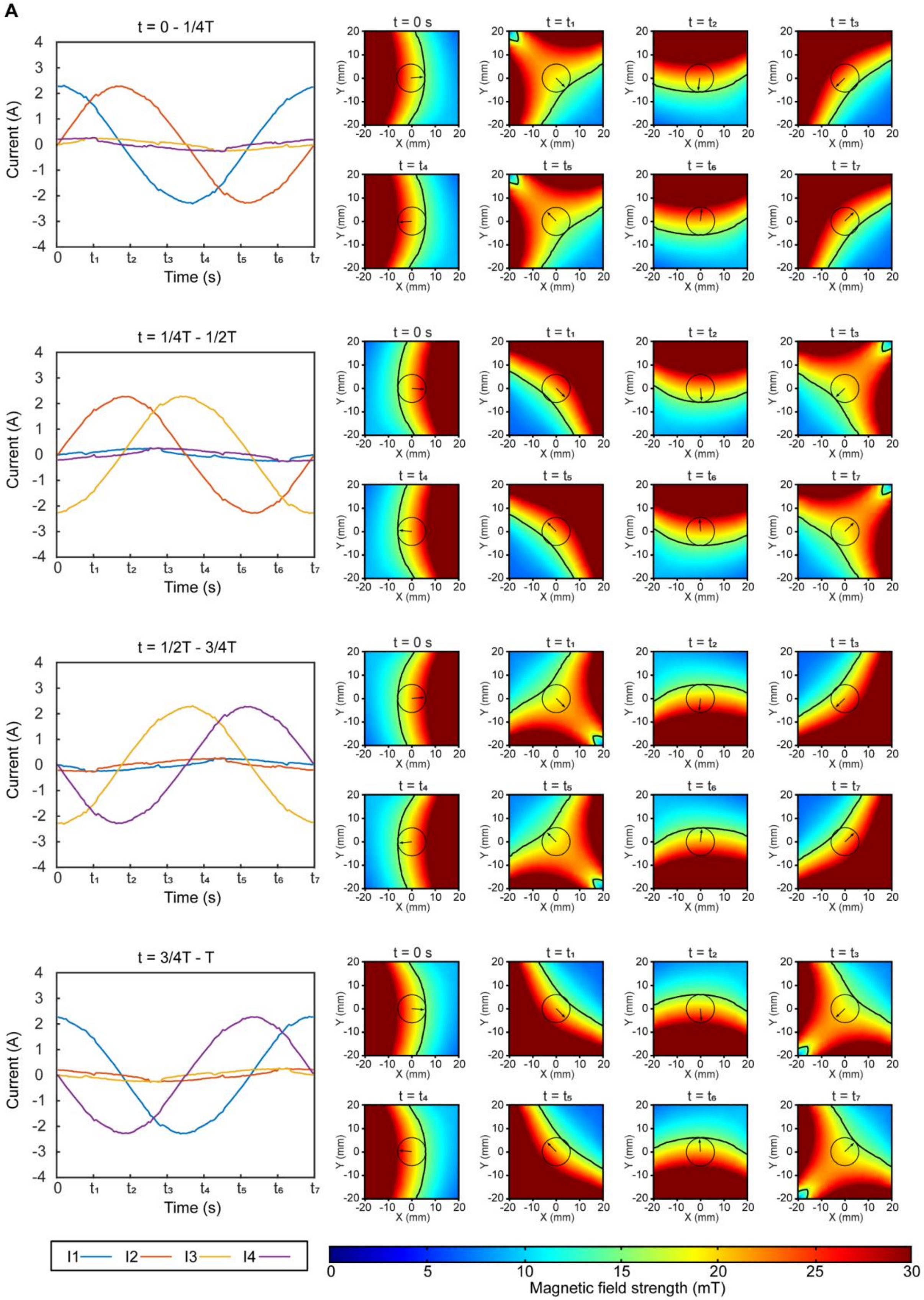
A
t = 0 - 1/4T
t = 1/4T - 1/2T
t = 1/2T - 3/4T
t = 3/4T - T
Current (A)
Time (s)
t = 0 s
t = t₁
t = t₂
t = t₃
t = t₄
t = t₅
t = t₆
t = t₇
Y (mm)
X (mm)
I1
I2
I3
I4
Magnetic field strength (mT)

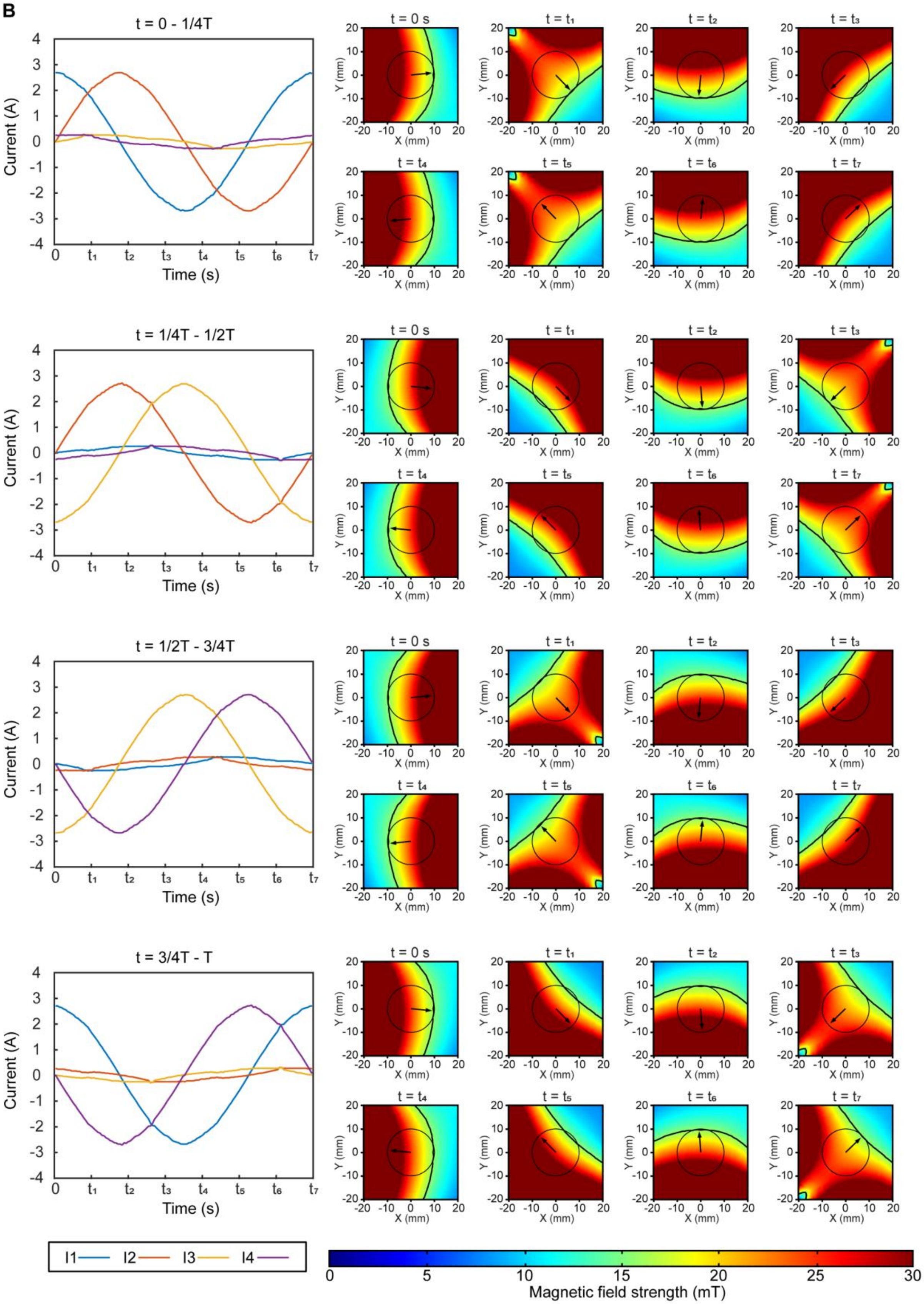
B
t = 0 - 1/4T
t = 1/4T - 1/2T
t = 1/2T - 3/4T
t = 3/4T - T
Current (A)
Time (s)
0 t₁ t₂ t₃ t₄ t₅ t₆ t₇
t = 0 s
t = t₁
t = t₂
t = t₃
t = t₄
t = t₅
t = t₆
t = t₇
X (mm)
Y (mm)
I1 I2 I3 I4
Magnetic field strength (mT)
0 5 10 15 20 25 30

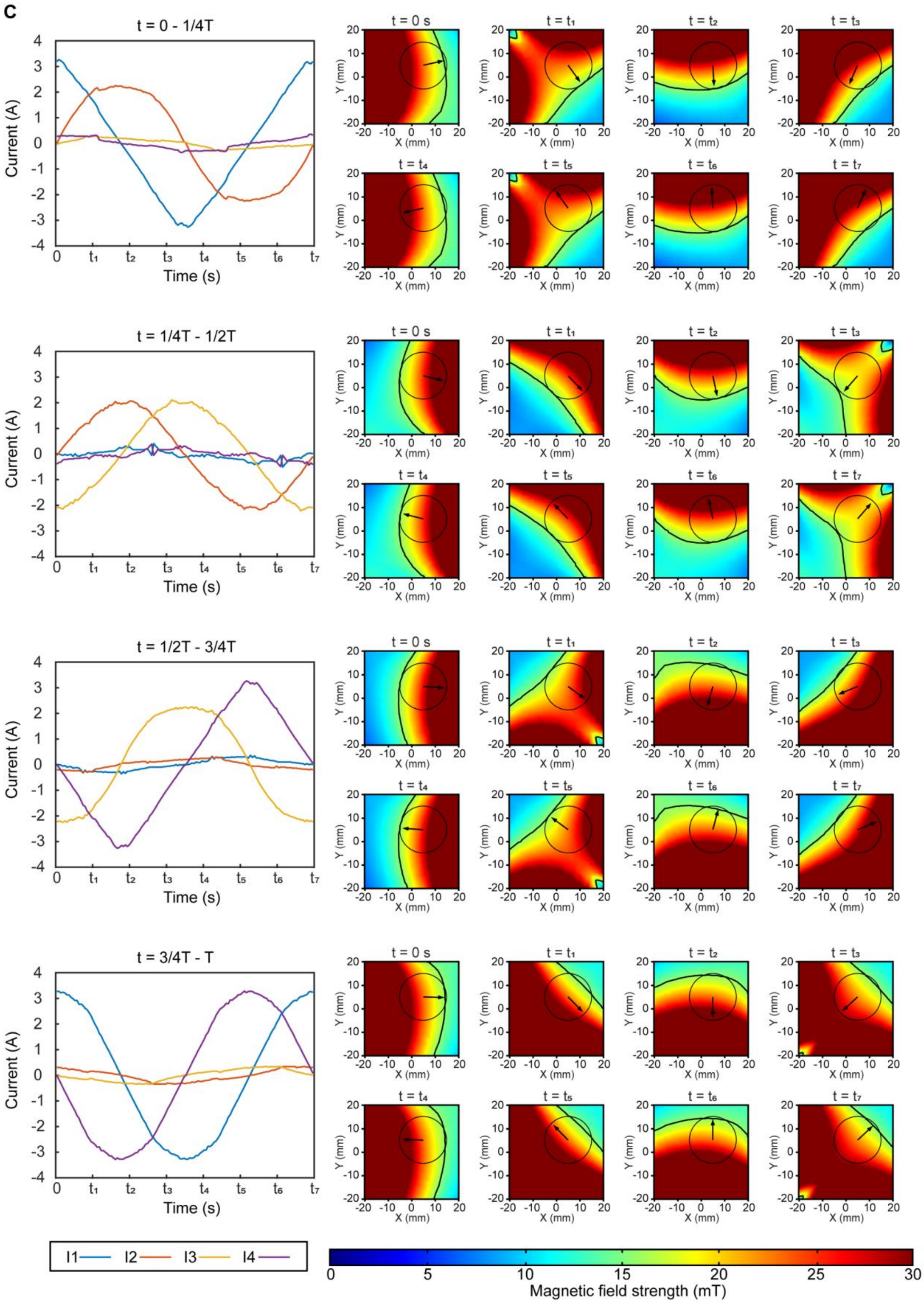
C
t = 0 - 1/4T
t = 1/4T - 1/2T
t = 1/2T - 3/4T
t = 3/4T - T
Current (A)
Time (s)
t = 0 s
t = t₁
t = t₂
t = t₃
t = t₄
t = t₅
t = t₆
t = t₇
X (mm)
Y (mm)
I1
I2
I3
I4
Magnetic field strength (mT)

Fig. S6. Simulation results of dynamic magnetic fields. (**A**, **B**, and **C**) Current sequences obtained from the brute-force search and the simulation results of dynamic magnetic fields in three cases, (A) P_{Q1} = (0 mm, 0 mm), r_{Q1} = 6 mm; (B) P_{Q2} = (0 mm, 0 mm), r_{Q2} = 10 mm; and (C) P_{Q3} = (5 mm, 5 mm), r_{Q3} = 10 mm. The black circles indicate the targeted regions. The black arrows indicate the direction of magnetic field. The black lines represent the fields where the strengths are equal to the critical magnetic field strengths $B_{critical}$ (16 mT in these cases).

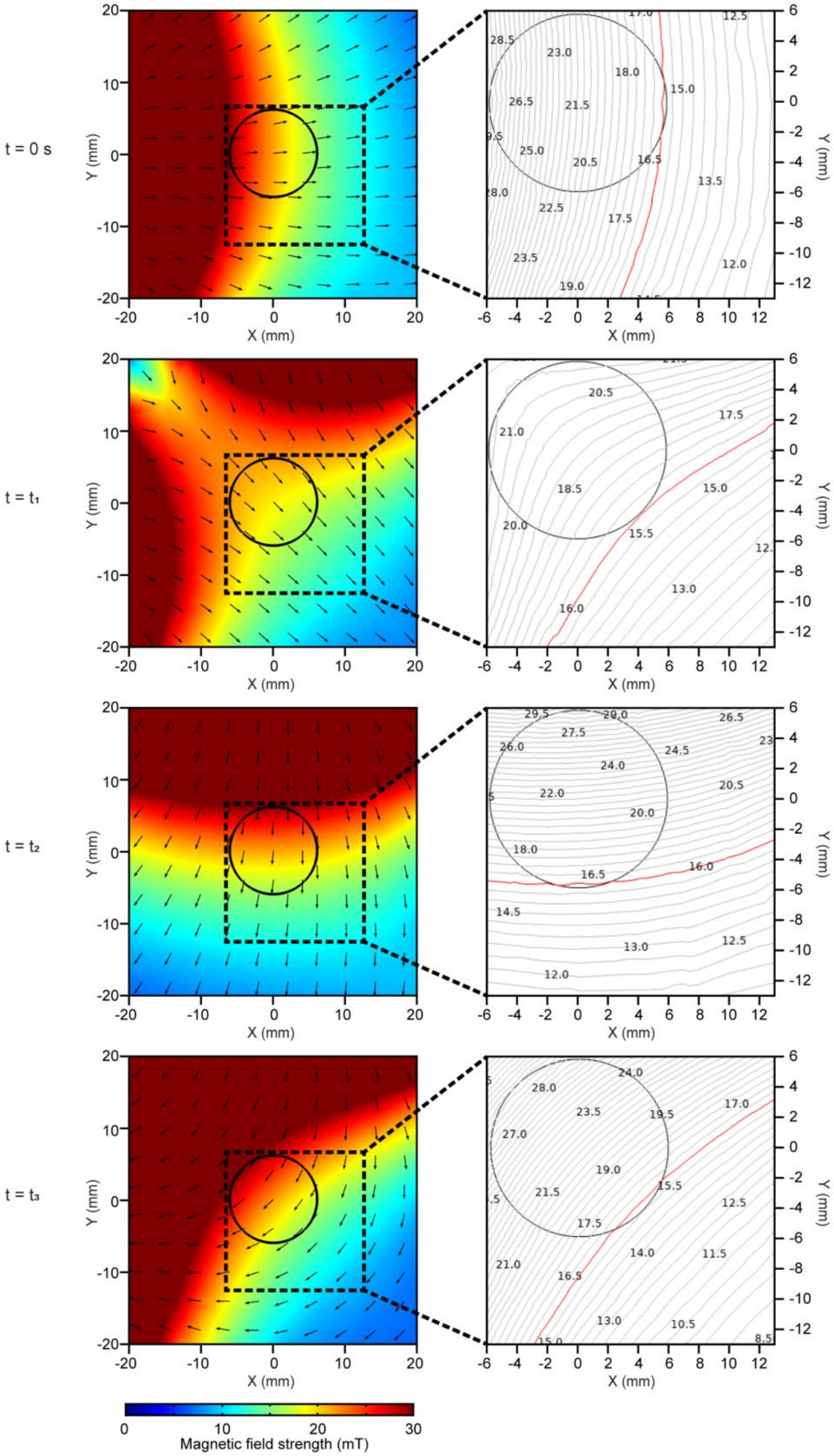

t = 0 s
t = t₁
t = t₂
t = t₃
X (mm)
Y (mm)
Magnetic field strength (mT)
0
10
20
30

Fig. S7. Zoomed-in views of simulation results of dynamic magnetic fields. The simulation results of dynamic magnetic fields in the case where P_{Q1} = (0 mm, 0 mm) and r_{Q1} = 6 mm. The black circles indicate the targeted regions. The black arrows indicate the direction of magnetic field vectors. The grey lines are scalar fields representing magnetic field strengths. The red lines represent the fields where the strengths are equal to the critical magnetic field strengths $B_{critical}$ (16 mT this case).

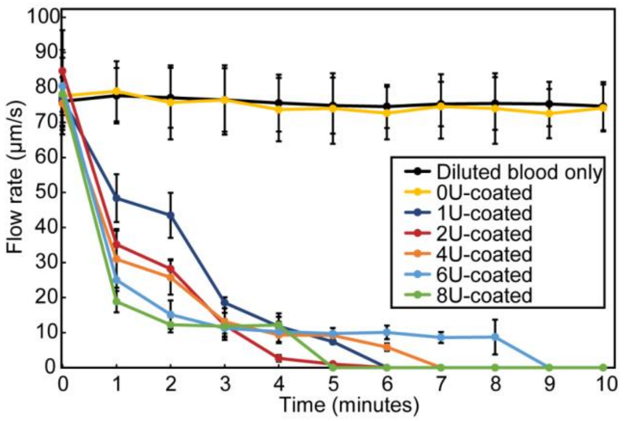

Fig. S8. Flow rate reduction by swarms composed of magnetic particles coated with different concentration of thrombin. The thrombin-coated magnetic particles were injected together with diluted porcine blood into microfluidic channels. 1U-coated indicates the magnetic particles were mixed with 1 U/mL thrombin solution during the coating process, and 2U-coated indicates 2 U/mL thrombin solution during the coating process. Each data point represents the average of ten measurements. Each measurement was made by measuring the speed of red blood cells. The error bars represent standard deviation.

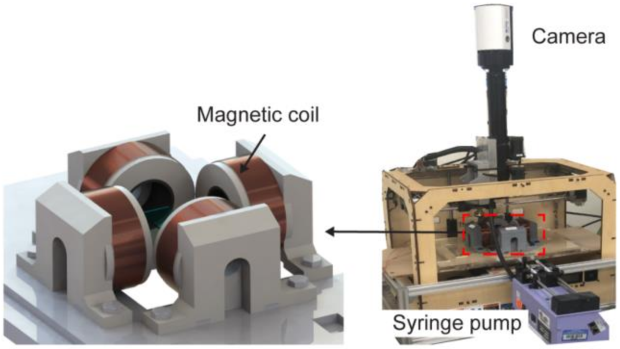

Fig. S9. Experimental setup. The system consists of four coils, a syringe pump, and a camera.

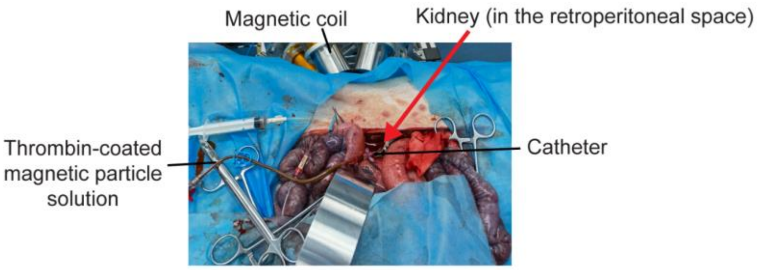

Fig. S10. ***In vivo* experimental setup.** The studies were conducted on porcine kidneys.

Table S1. Data for the estimations of magnetic dipole-dipole interaction force, van der Waals force, and electrostatic force.

Particle magnetic susceptibility	x_p	0.58
Free space permeability	μ_0	1.257×10^{-6} [kg m s^{-2} A^{-2}]
Magnetic field strength	B	10×10^{-3} [T]
Hamaker constant	A_h	20×10^{-20} [J]
Particle radius	a_p	0.5×10^{-6} [m]
Free space permittivity	ε_0	8.85×10^{-12} [C^2 J^{-1} m^{-1}]
Relative blood permittivity	ε_p	76.8
Boltzmann constant	k_B	1.38×10^{-23} [J K^{-1}]
Temperature	T	310.15 [K]
Elementary electric charge	e	1.6×10^{-19} [C]
Particle surface potential	φ_p	20 [mV]
Avogadro's number	N_A	6.02×10^{23} [mol^{-1}]
Blood ionic strength	I	150 [mol m^{-3}]
Separation distance	h	0.1×10^{-6} [m]

Table S2. Comparisons between the predictions of 2D swarm integrity model and those of 3D swarm integrity models at different branching angles.

	Branching angle	30 degrees	60 degrees	90 degrees	120 degrees
P-value	2D model and 3D model I	0.49	0.47	0.47	0.49
	2D model and 3D model II	0.36	0.43	0.40	0.19
	2D model and 3D model III	0.17	0.36	0.17	0.22

Table S3. Relative errors of the models.

Model	Relative error (%)	
	Blood plasma	PBS solution
2D model	8.0	9.1
3D model I	7.9	9.1
3D model II	7.8	9.0
3D model III	8.1	9.9

Supplementary Note 1 – Three-dimensional swarm models

The neighboring particles of tip-particles are indexed in Fig. S3a. For the model based on configuration I (3D model I in Fig. S3), the angles between the dipole-dipole magnetic interaction force vectors $\mathbf{F_{int,ij}}$ (exerted by neighboring particles) and the XY plane are $\alpha_1 \approx -\frac{\pi}{4}$, $\alpha_2 \approx -\frac{\pi}{4}$, $\alpha_3 \approx -\frac{\pi}{2}$, $\alpha_4 \approx -\arctan\left(\left(2\sin\left(\frac{\pi}{6}+\frac{\gamma}{3}\right)\right)^{-1}\right)$, $\alpha_5 \approx 0$, $\alpha_6 \approx 0$, $\alpha_7 \approx 0$, $\alpha_8 \approx \frac{\pi}{4}$, $\alpha_9 \approx \frac{\pi}{4}$, $\alpha_{10} \approx \frac{\pi}{2}$, and $\alpha_{11} \approx \arctan\left(\left(2\sin\left(\frac{\pi}{6}+\frac{\gamma}{3}\right)\right)^{-1}\right)$. The angles of the projections of vectors $\mathbf{F_{int,ij}}$ in the XY plane with respect to the X-axis are $\beta_1 \approx \frac{\pi}{6}-\frac{\gamma}{3}$, $\beta_2 \approx -\frac{\pi}{6}-\frac{\gamma}{3}$, $\beta_3 \approx 0$, $\beta_4 \approx -\frac{\pi}{2}$, $\beta_5 \approx \frac{\pi}{6}-\frac{\gamma}{3}$, $\beta_6 \approx -\frac{\pi}{6}-\frac{\gamma}{3}$, $\beta_7 \approx -\frac{\pi}{2}$, $\beta_8 \approx \frac{\pi}{6}-\frac{\gamma}{3}$, $\beta_9 \approx -\frac{\pi}{6}-\frac{\gamma}{3}$, $\beta_{10} \approx 0$, and $\beta_{11} \approx -\frac{\pi}{2}$. The distances between the centers of the neighboring particles and that of the tip-particle are $r_1 \approx \sqrt{2}d$, $r_2 \approx \sqrt{2}d$, $r_3 \approx d$, $r_4 \approx \sqrt{d^2+\left(2d\sin\left(\frac{\pi}{6}+\frac{\gamma}{3}\right)\right)^2}$, $r_5 \approx d$, $r_6 \approx d$, $r_7 \approx 2d\sin\left(\frac{\pi}{6}+\frac{\gamma}{3}\right)$, $r_8 \approx \sqrt{2}d$, $r_9 \approx \sqrt{2}d$, $r_{10} \approx d$, and $r_{11} \approx \sqrt{d^2+\left(2d\sin\left(\frac{\pi}{6}+\frac{\gamma}{3}\right)\right)^2}$. The model calibration factor was calculated to be 2.6 based on the experimental results in Fig. 2a,b.

For the model based on configuration II (3D model II in Fig. S3), the angles between the dipole-dipole magnetic interaction force vectors $\mathbf{F_{int,ij}}$ and the XY plane are $\alpha_1 \approx 0$, $\alpha_2 \approx -\arctan\left(\frac{1}{2}\right)$, $\alpha_3 \approx 0$, and $\alpha_4 \approx \arctan\left(\frac{1}{2}\right)$. The angles of the projections of vectors $\mathbf{F_{int,ij}}$ in the XY plane with respect to the X-axis are $\beta_1 \approx \frac{\pi}{6}-\frac{\gamma}{3}$, $\beta_2 \approx -\frac{\pi}{6}-\frac{\gamma}{3}$, $\beta_3 \approx -\frac{\pi}{2}$ and $\beta_4 \approx -\frac{\pi}{6}-\frac{\gamma}{3}$. The distances between the centers of the neighboring particles and that of the tip-particle are $r_1 \approx d$, $r_2 \approx \sqrt{\frac{5}{4}}d$, $r_3 \approx 2d\sin\left(\frac{\pi}{6}+\frac{\gamma}{3}\right)$, and $r_4 \approx \sqrt{\frac{5}{4}}d$. The model calibration factor was calculated to be 2.6 based on the experimental results in Fig. 2a,b.

For the model based on configuration III (3D model III in Fig. S3), the angles between the dipole-dipole magnetic interaction force vectors $\mathbf{F_{int,ij}}$ and the XY plane are $\alpha_1 \approx 0$, $\alpha_2 \approx -\arctan\left(\frac{1}{2}\right)$, $\alpha_3 \approx -\arctan\left(\frac{1}{2}\right)$, $\alpha_4 \approx 0$, $\alpha_5 \approx \arctan\left(\frac{1}{2}\right)$, and $\alpha_6 \approx \arctan\left(\frac{1}{2}\right)$. The angles

of the projections of vectors $\mathbf{F}_{\mathbf{int,ij}}$ in the XY plane with respect to the X-axis are $\beta_1 \approx \frac{\pi}{3} - \frac{\gamma}{3}$, $\beta_2 \approx \frac{\pi}{6} - \frac{\gamma}{3}$, $\beta_3 \approx -\frac{\pi}{6} - \frac{\gamma}{3}$, $\beta_4 \approx -\frac{\pi}{2}$, $\beta_5 \approx -\frac{\gamma}{2}$, and $\beta_6 \approx -\frac{\pi}{4} - \frac{\gamma}{4}$. The distances between the centers of the neighboring particles and that of the tip-particle are $r_1 \approx d$, $r_2 \approx \sqrt{\frac{5}{4}}d$, $r_3 \approx \sqrt{\frac{5}{4}}d$, $r_4 \approx 2d \sin\left(\frac{\pi}{6} + \frac{\gamma}{3}\right)$, $r_5 \approx \sqrt{\frac{5}{4}}d$, and $r_6 \approx \frac{3}{2}d$. The model calibration factor was calculated to be 2.6 based on the experimental results in Fig. 2a,b.

Movie S1. Splitting of swarms occurs when the applied magnetic field strength was lower than $B_{critical}$. The branching angle of the junction was 120° and the flow was injected at 80 µm/s. $B_{critical}$ was calculated to be 16 mT and the magnetic field strength applied was 15 mT.

Movie S2. Maintenance of swarm integrity at a junction. The branching angle of the junction was 120° and the flow was injected at 80 µm/s. $B_{critical}$ was calculated to be 16 mT and the magnetic field strength applied was 20 mT.

Movie S3. Simulation results of dynamic magnetic fields, with the current sequences obtained from the brute-force search. The brown and white coils are dominant and auxiliary coils, respectively. The black circles indicate the targeted regions. The black arrows indicate the direction of magnetic field. The black lines link the points with magnetic field strength equal to $B_{critical}$, 16 mT in this case.

Movie S4. Experimental results using thrombin-coated magnetic particles to occlude a branched channel. The branching angle of the junction was 60° and the flow was injected at 80 µm/s. $B_{critical}$ was calculated to be 19 mT and a 20 mT uniform rotating magnetic field was applied.

Movie S5. *Ex vivo* demonstration of selective embolization in a porcine omentum using our proposed strategy. The branching angle of the targeted vessel was approximately 100° and the flow was injected at 80 µm/s. $B_{critical}$ was calculated to be 20 mT and a brute-force search-generated dynamic magnetic field was applied.
